# Dynamic Gut Microbiome Changes in Response to Low-Iron Challenge

**DOI:** 10.1128/AEM.02307-20

**Published:** 2021-01-15

**Authors:** Genevieve L. Coe, Nicholas V. Pinkham, Arianna I. Celis, Christina Johnson, Jennifer L. DuBois, Seth T. Walk

**Affiliations:** a Department of Chemistry and Biochemistry, Montana State University, Bozeman, Montana, USA; b Department of Microbiology and Immunology, Montana State University, Bozeman, Montana, USA; University of Bayreuth

**Keywords:** gut microbiome, host response, iron metabolism, microbial ecology

## Abstract

Iron is an essential micronutrient for life. In mammals, dietary iron is absorbed primarily in the small intestine. Currently, the impacts of dietary iron on the taxonomic structure and function of the gut microbiome and reciprocal effects on the animal host are not well understood. Here, we establish a mouse model of low-iron challenge in which intestinal biomarkers and reduced fecal iron reveal iron stress while serum iron and mouse behavioral markers indicate maintenance of iron homeostasis. We show that the diversity of the gut microbiome in conventional C57BL/6 mice changes dramatically during 2 weeks on a low-iron diet. We also show the effects of a low-iron diet on microbiome diversity are long lasting and not easily recovered when iron is returned to the diet. Finally, after optimizing taxon association methods, we show that some bacteria are unable to fully recover after the low-iron challenge and appear to be extirpated from the gut entirely. In particular, operational taxonomic units (OTUs) from the *Prevotellaceae* and *Porphyromonadaceae* families and *Bacteroidales* order are highly sensitive to low-iron conditions, while other seemingly insensitive OTUs recover. These results provide new insights into the iron requirements of gut microbiome members and add to the growing understanding of mammalian iron cycling.

**IMPORTANCE** All cells need iron. Both too much and too little iron lead to diseases and unwanted outcomes. Although the impact of dietary iron on human cells and tissues has been well studied, there is currently a lack of understanding about how different levels of iron influence the abundant and diverse members of the human microbiome. This study develops a well-characterized mouse model for studying low-iron levels and identifies key groups of bacteria that are most affected. We found that the microbiome undergoes large changes when iron is removed from the diet but that many individual bacteria are able to rebound when iron levels are changed back to normal. That said, a select few members, referred to as iron-sensitive bacteria, seem to be lost. This study begins to identify individual members of the mammalian microbiome most affected by changes in dietary iron levels.

## Abstract (precis)

All cells need iron. Both too much and too little iron lead to diseases and unwanted outcomes.

## INTRODUCTION

Iron is an essential nutrient for nearly all life, mediating numerous biochemical reactions involving dioxygen (O_2_) and atom or electron transfers (1). In mammals, these include the transport of oxygen to tissues via iron protoporphyrin IX (heme) bound by hemoglobin in red blood cells, respiratory electron transfer (cytochrome *c* and cytochrome *c* oxidase), and degradation of hydrogen peroxide (catalase) (2). Anemia is an extremely common metabolic disorder worldwide, caused either by a failure to metabolize iron correctly or by a lack of sufficient iron in the diet (3). While several human and animal nutritional studies have examined the systemic effects of dietary iron deficiency (4), how iron availability influences gut microbiome diversity and taxonomic structure, and whether some taxa are more sensitive than others to diets containing low levels of iron, is unclear.

To address this knowledge gap, we conducted a series of experiments using conventional C57BL/6 mice to identify the most iron-sensitive microbiome members. We first established a model for low-iron (LI) challenge that includes acclimatization to a chemically defined diet, itself a major perturbance to the microbiome, prior to evaluating the impact of LI challenge on both α- and β-diversity using 16S rRNA sequence analysis. Finally, we evaluated bioinformatic selection procedures for identifying iron-sensitive taxa and quantified their ability to recover after LI challenge when dietary iron was restored. The model described here incorporates an acclimatization period into a murine model of microbiome-iron interaction and, therefore, serves as a benchmark for understanding the influence of this essential mineral on microbiome structure and function.

## RESULTS

### Establishment of LI challenge model.

**FIG 1:**
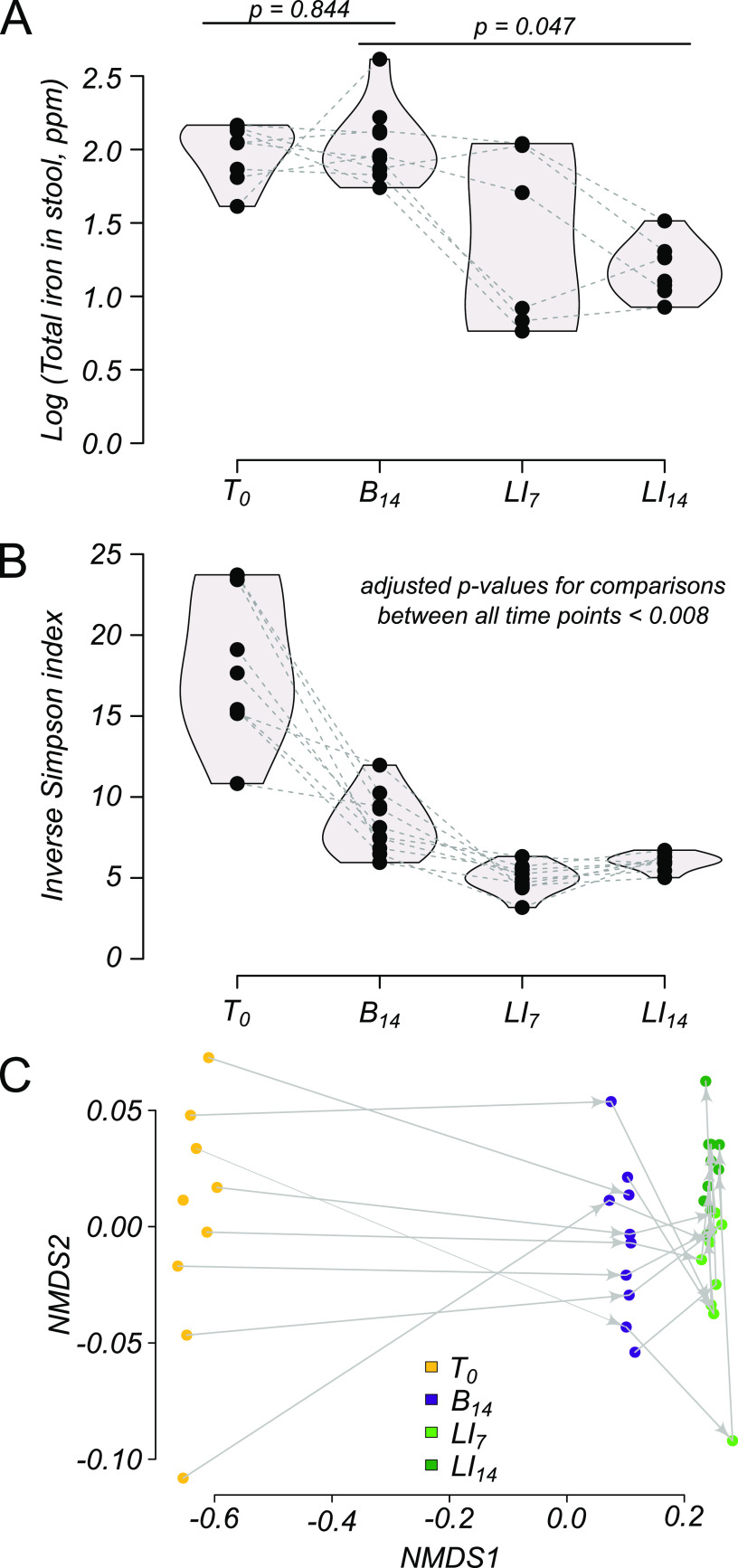
Microbiome response to diet shift/LI challenge. (A) A pilot experiment was conducted to quantify total iron (log_10_ scale) in stool pellets at baseline (B_14_) (see the text) and after one (LI_7_) and two (LI_14_) weeks of LI challenge. Lines connect individual animals throughout time if data were available (data were not available for all mice at all time points; see Materials and Methods) (B) α-Diversity (inverse Simpson’s index) of the microbiome at baseline and 2 weeks of LI challenge. (C) Nonmetric multidimensional scaling of β-diversity (Bray-Curtis dissimilarity) at baseline and 2 weeks of LI challenge. All *P* values represent paired (nonparametric) Wilcoxon rank sum tests with Benjamini-Hochberg correction.

To evaluate the impact of a low-iron (LI) diet on gut microbiome diversity, we developed a feeding protocol in conventional, specific-pathogen-free mice where stool samples and blood were taken throughout dietary transitions. The typical chow used in our vivarium contained 208 mg total iron/kg of body weight (quantified by inductively coupled plasma-mass spectrometry [ICP-MS]), primarily as ferrous (Fe^2+^) carbonate. Purified chow containing iron at 2.6 ppm (in milligrams of total iron per kilogram, quantified by ICP-MS) was purchased for use as an LI challenge. Since other nutritional components of the LI diet were markedly different from typical mouse chow, we first conducted a pilot study to establish a new baseline (B) of microbiome diversity for comparison. Namely, we switched the diet of conventional mice to the LI challenge chow and added ferrous iron [iron(II) sulfate heptahydrate] to drinking water at a level that maintained a similar level of iron in the gut (Fig. 1A).

In the initial experiment, we found that mice eating the LI diet with iron supplemented in their drinking water for 2 weeks (time zero [T_0_] to baseline day 14 [B_14_]) maintained the same level of iron in their stool as they did when eating normal chow, without statistical differences (*P* = 0.844 by paired Wilcoxon rank sum test) (Fig. 1A). This indicated that this level of iron supplementation (0.32 mM Fe) was sufficient to replicate standard chow. Interestingly, we noticed that there was a significant decrease in microbiome α-diversity from only changing chow diet and water (*P* = 0.006 by paired Wilcoxon rank sum test) (Fig. 1B). A significant β-diversity shift (f = 60.3, *P* = 0.001 by permutational analysis of variance [PERMANOVA]) occurred and was comparable for the cohort (Fig. 1C, shift in yellow to purple). These results supported our expectation that switching the murine diet (normal to the chemically defined LI chow) had a significant impact on microbiome composition even though the mice were exposed to similar levels and types of iron. This suggests that diet transitions must be stabilized before observing the effects on the dietary variable.

The removal of FeSO_4_ from the drinking water resulted in a significant decrease in total iron in stool samples over a 2-week time frame (*P* = 0.047 by Wilcoxon rank sum test) (Fig. 1A, B_14_ to LI_14_), indicating that our iron challenge protocol was sufficient for lowering the amount of iron in the gut. Concomitant with low iron conditions in the stool, we saw a further decrease in population richness in α-diversity (Fig. 1B) and another significant β-diversity shift (Fig. 1C), indicating a substantial microbiome response to iron challenge. Furthermore, effect sizes of β-diversity shifts between time points varied throughout the LI challenge protocol. Importantly, the most drastic change in both measures of diversity occurred over the first 7 days of iron limitation from B_14_ to LI_7_, suggesting that the microbiome stabilized after the first week of the LI challenge (LI_7_), where relatively little change was observed after the second week (LI_14_). Collectively, the results from this initial experimental setup suggest that (i) our protocol established a reasonable baseline following the dietary shift and (ii) the microbiome responded to the LI challenge.

### Reproducible microbiome dynamics and host response during LI challenge.

**FIG 2:**
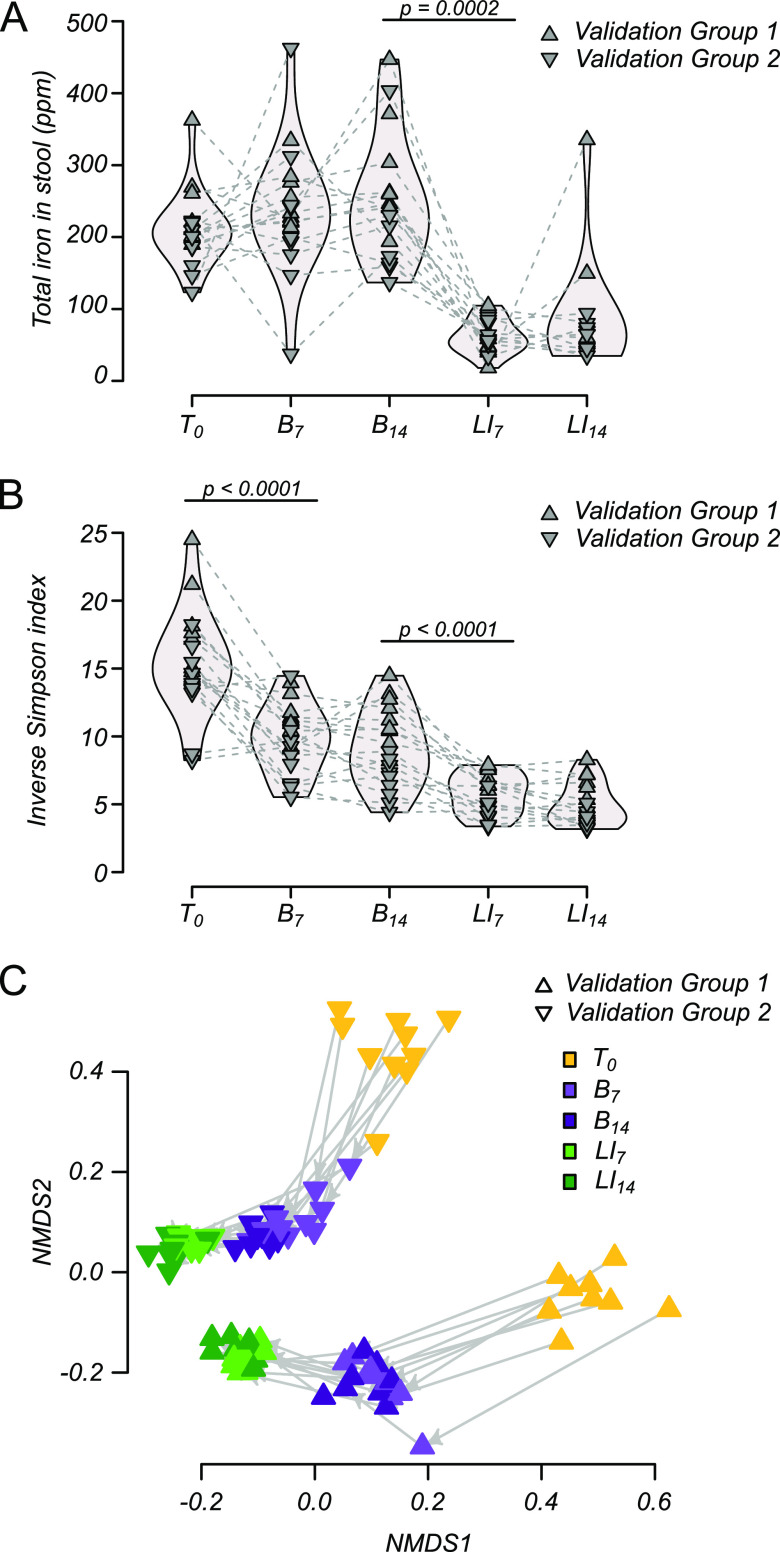
Reproducible microbiome response to diet shift/LI challenge. (A) Total iron in stool pellets of two validation groups of mice on normal chow (T_0_), throughout 2 weeks of baseline (B_7_ and B_14_), and 2 weeks of LI challenge (LI_7_ and LI_14_). (B) α-Diversity (inverse Simpson’s index) of the microbiome throughout diet shift/LI challenge. (C) Nonmetric multidimensional scaling of β-diversity (Bray-Curtis dissimilarity) throughout diet shift/LI challenge. All *P* values represent paired (nonparametric) Wilcoxon rank sum tests with Benjamini-Hochberg correction, and only significant differences between subsequent time points are shown.

To evaluate the reproducibility of microbiome dynamics between experiments and to assess markers of host response to iron, two “validation” experiments were conducted using the LI challenge protocol described above. In these experiments, stool samples were taken at an additional baseline time point (B_7_) to better assess microbiome stability during baseline establishment. Consistent with results from the pilot experiment, total iron in stool did not change significantly between T_0_ and baseline time point B_7_ or B_14_ but decreased significantly in response to the LI challenge (B_14_ to LI challenge day 7 [LI_7_]; *P* = 0.0002 by paired Wilcoxon rank sum test) (Fig. 2A). Stool iron levels stabilized during the LI challenge and did not change significantly between LI_7_ and LI_14_.

During interval transition from vivarium chow to LI chow, there was a decrease in α-diversity (Fig. 2B). This was similar to the pilot experiment where iron supplementation in the water was removed and demonstrates the robust application of this protocol to diversity measures in these stages. No differences were observed between baseline (B_7_ and B_14_) or LI challenge time points (LI_7_ and LI_14_), suggestive of microbiome stability during both of these intervals. Surprisingly, we found that the microbiome of these two groups of mice were markedly different at the outset of each experiment (T_0_), potentially reflecting vivarium-specific differences in microbiome diversity even when exposed to similar housing and feeding conditions (see Fig. S2 in the supplemental material for experiment-wise diversity estimates). Regardless of the initial starting differences, similar changes in ordination space occurred throughout the LI challenge phase (Fig. 2C). These results suggest that the LI challenge produces highly reproducible microbiome effects even in the face of experiment-to-experiment and mouse cohort variation. The described protocol reproduced results providing robustness in the microbiome response regardless of the initial composition of diversity in the host.

**FIG 3:**
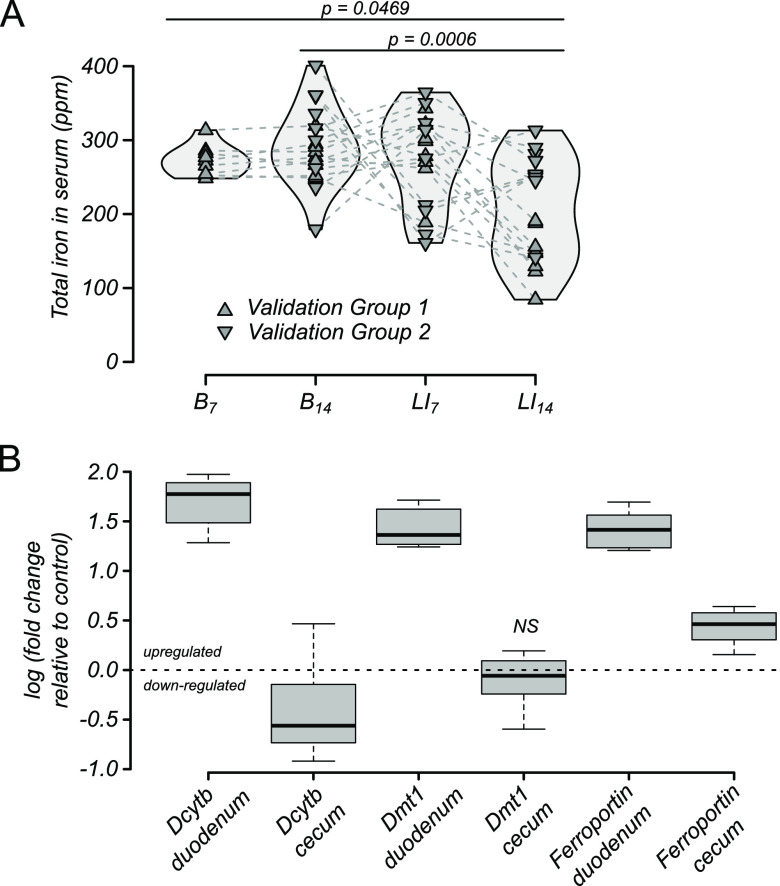
Host response to LI challenge. (A) Total serum iron in mice throughout baseline (B_7_ and B_14_) and LI challenge (LI_7_ and LI_14_) in mice shown in Fig. 2. All *P* values represent paired (nonparametric) Wilcoxon rank sum tests with Benjamini-Hochberg correction. (B) Iron-responsive gene expression in mice after 2 weeks of LI challenge. The only nonsignificant change in gene expression compared to control mice (*P* > 0.05 by *t* test) was Dmt1 in cecal tissue.

To focus on host absorption of iron and metabolic response, changes in serum iron were quantified from blood drawn weekly from all mice. LI challenge decreased host iron stores when total iron levels were quantified in serum throughout the model timeline (Fig. 3A). No significant differences were observed between baseline time points B_7_ and B_14_ or between baseline and the first week of LI challenge (LI_7_). However, total serum iron decreased from baseline in mice during the second week of LI challenge (B_7_ and B_14_ versus LI_14_), suggesting host iron stores were being drawn down. Over the same time intervals, stool iron decreased, indicating that a host metabolic response led to recycling and retention rather than excretion of systemic iron. Duodenal tissue collected at the end of the LI challenge and assayed for iron-responsive gene expression supported the proposed explanation of iron recycling and retention, as all three genes of interest were significantly upregulated (Fig. 3B). In contrast, no consistent effect on gene expression was observed in cecal tissue, which was used as a type of internal negative control because the same genes are known to be much less responsive to iron in the cecum compared to the duodenum.

**FIG 4:**
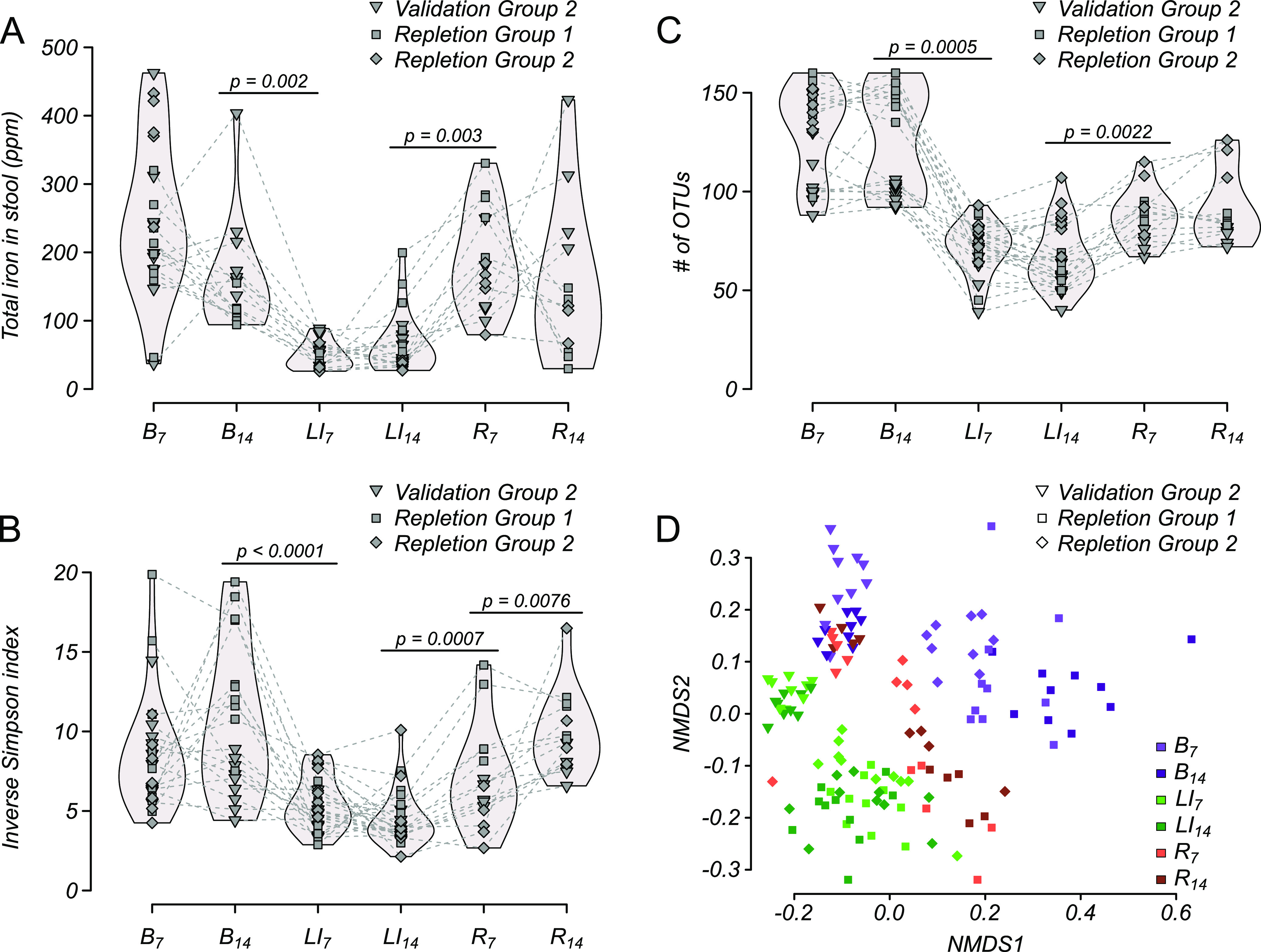
Microbiome response to LI challenge and iron repletion. (A) Total iron in stool pellets of validation group 2 and two additional groups of mice (repletion groups 1 and 2; *n* = 10 each) throughout 2 weeks of baseline (B_7_ and B_14_), 2 weeks of LI challenge (LI_7_ and LI_14_), and 2 weeks on the same diet but with iron levels repleted (R_7_ and R_14_). (B) α-Diversity (inverse Simpson’s index) of the microbiome throughout LI challenge/repletion. (C) Microbiome richness (number of OTUs) throughout LI challenge/repletion. (D) Nonmetric multidimensional scaling of β-diversity (Bray-Curtis dissimilarity) throughout LI challenge/repletion. All *P* values represent paired (nonparametric) Wilcoxon rank sum tests with Benjamini-Hochberg correction, and only significant differences between subsequent time points are shown.

For one of the validation experiments (validation group 2) as well as for two iron “repletion” experiments (repletion groups 1 and 2) that received iron repletion for 2 weeks following LI challenge (R_14_), no change in host serum iron was evident either during LI challenge or repletion (Fig. 4A), again suggesting that mice had not yet depleted their iron stores. Consistent with this result, the increases in iron-responsive gene expression of Dcybt, DMT1, and ferroportin seen in duodenal tissue in validation group 1 (Fig. 3B) was not observed by the end of the repletion phase (Fig. 4B). Collectively, these results suggest that mice responded to the LI challenge by upregulating gut transport pathways for iron but were not stressed to the point of stably lowering systemic circulating iron in the serum, as seen in physiological anemia.

### LI challenge has long-lasting microbiome effects.

**FIG 5:**
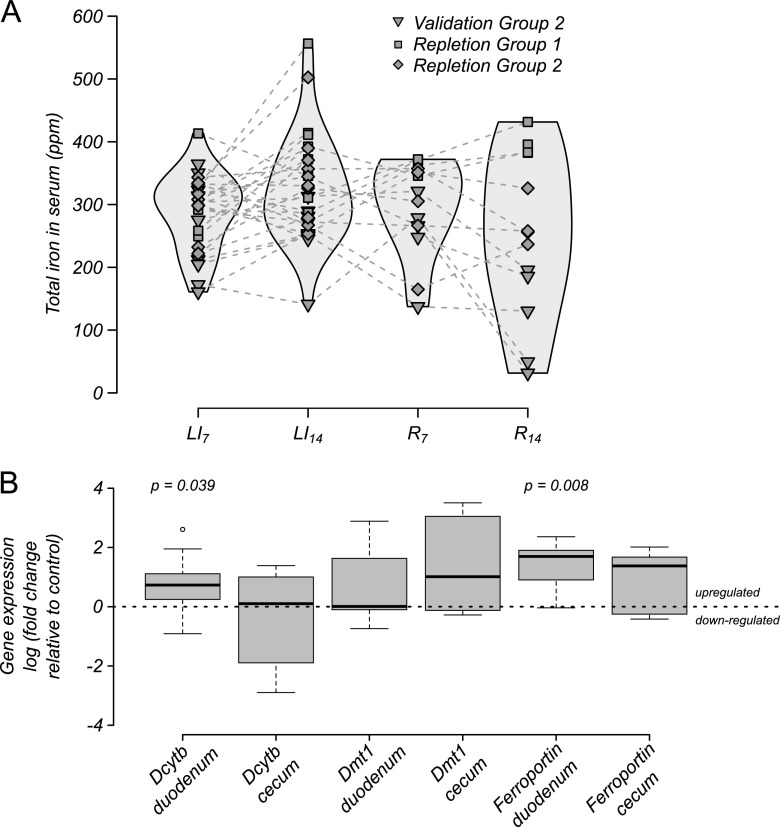
Host response to LI challenge and iron repletion. (A) Total serum iron in mice throughout LI challenge (LI_7_ and LI_14_) and 2 weeks on the same diet but with iron levels repleted (R_7_ and R_14_). No significant changes were observed. (B) Iron-responsive gene expression in mice after 2 weeks with iron repletion. All changes were nonsignificant (*P* > 0.05 by *t* test) except for duodenal Dcytb and ferroportin, as indicated.

As mentioned above, we conducted three independent experiments (validation group 2 and repletion groups 1 and 2) to understand whether alteration of microbiome diversity as a result of LI challenge would last when iron was reintroduced as opposed to simply shifting back to baseline (i.e., are changes in microbiome diversity due to low iron levels ameliorated when iron was added back?). In these experiments, a decrease in stool iron was observed between baseline and LI challenge time points (Fig. 5A), similar to results in Fig. 1A and 2A. The drop in total stool iron was reversed in the first week of repletion (R_7_) and was comparable throughout the second week of repletion (R_14_). Similarly, decreased α-diversity was observed in all groups between baseline and LI time points but was significantly reversed during the first week of repletion (Fig. 5B). Interestingly, α-diversity did not entirely stabilize and was significantly greater after the second week of repletion compared to the first (increased from R_7_ to R_14_). Not all components of microbiome diversity were affected equally during this 2-week repletion period. For example, there was no difference in the total number of observed operational taxonomic units (OTUs) (i.e., richness) during time points R_7_ to R_14_ (Fig. 5C), but OTU relative abundance (i.e., evenness) did not recover to the prechallenge levels during the 2 weeks of iron repletion. Nonmetric multidimensional scaling (NMDS) plots of β-diversity shown in Fig. 5D also supported that recovery was not complete during repletion, as microbiome diversity at time point R_14_ was significantly different from baseline (B_7_) in all groups (by PERMANOVA; validation group 2, *P* = 0.001, pseudo-F = 5.38; repletion group 1, *P* = 0.001, pseudo-F = 10.73; repletion group 2, *P* = 0.002, pseudo-F = 9.18). Incomplete rescue of both α- and β-diversity estimates suggests that LI challenge had long-lasting effects on microbiome diversity even after stool iron levels returned to normal.

### Specific OTUs are affected by LI challenge.

Few comparisons of the performance of OTU-picking methods have been conducted, meaning there is currently little consensus in the field about which approach is most appropriate for identifying OTUs most affected by a given treatment. Here, we compared and evaluated four different approaches (random forest analysis, indicator value analysis, OTU presence-absence, and multiple *t* testing with Bonferroni correction) based on straightforward OTU selection criteria.

**FIG 6:**
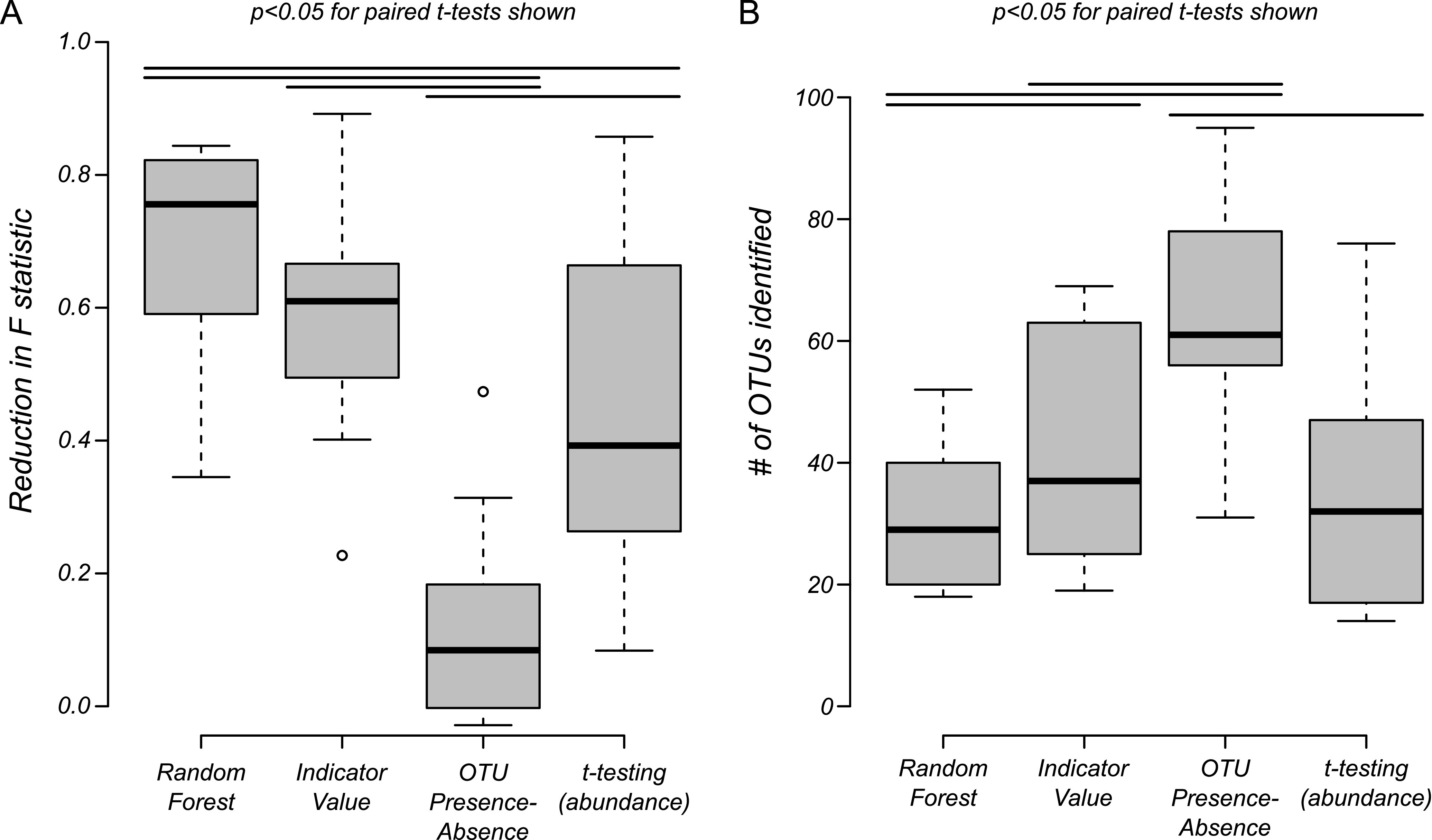
OTU selection technique performance. Four techniques were compared for selecting OTUs most affected throughout experimental treatment transitions (T_0_ to B_7_, B_14_ to LI_7_, and LI_14_ to R_7_). Box plots represent median and interquartile ranges (whiskers indicate the range). Lines above boxes represent significant differences (all *P* values of  <0.05) from paired *t* testing (paired by experimental group; see Tables S2 and S3). Random forest analysis yielded the greatest reduction in PERMANOVA pseudo-F statistic (A) and identified the fewest number of OTUs (B) across all transitions.

**FIG 7:**
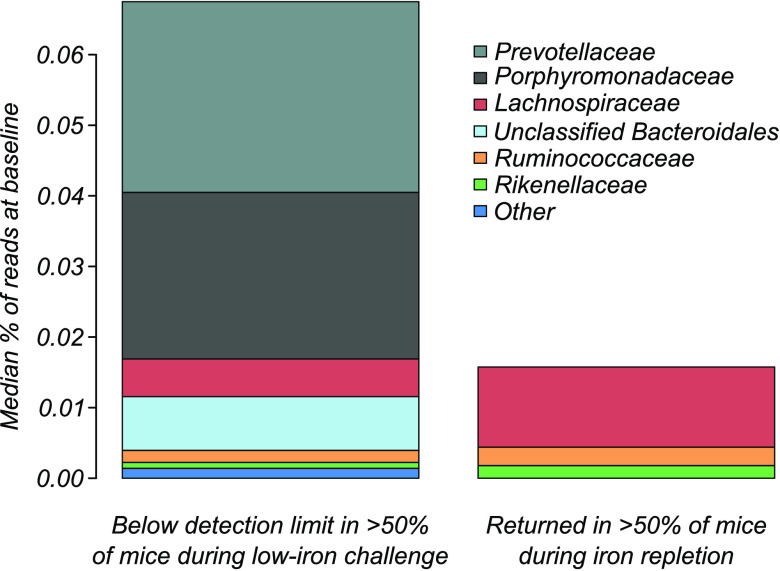
OTUs most affected by LI challenge. Random forest analysis identified specific OTUs most affected by LI challenge. These OTUs were present at appreciable amounts at baseline, fell below the detection limit during LI challenge (left), and were either able to recover (right) or remained undetectable.

Results generated for each group at the major dietary treatment transitions (T_0_ to B_7_, B_14_ to LI_7_, and LI_14_ to R_7_) were compared (Fig. 6 and Tables S2 and 3). We found that random forest analysis performed the best overall by giving the greatest reduction of the PERMANOVA test statistic (Fig. 6A) while identifying the lowest number of OTUs (Fig. 6B). OTUs identified by random forest analysis, selected across all time points and for each experimental group, are listed in Table S4. Of the 310 OTUs in the microbiome of all mice in validation group 2/repletion groups 1 and 2, random forest analysis identified six that were present at appreciable levels at baseline and undetectable in more than half of the mice during LI challenge (Fig. 7). Of the six identified, three OTUs belonging to *Lachnospiraceae*, *Ruminococcaceae*, and *Rikenellaceae* families were recovered with iron repletion, whereas OTUs classified as *Prevotellaceae*, *Porphyromonadaceae*, and an unclassified *Bacteroidales* remained undetectable and presumably were not recovered.

## DISCUSSION

Iron is an essential nutrient for mammals and the diverse community of microbes inhabiting the mammalian gastrointestinal (GI) tract. The taxonomic structure of the gut microbiome is known to be sensitive to the chemical speciation and overall amount of iron in the host’s diet. For example, feeding mice a heme-based diet was shown to result in an increased proportion of *Bacteroides* species in stool (5, 6). Recent work further demonstrated that in an antibiotic-attenuated microbiome, *Lactobacillus* species sense iron deprivation and communicate iron need to a murine host via the production of small-molecule mediators that interact with the Fe/O_2_-responsive transcription factor, HIF-2α (7). Such studies support the overall hypothesis that certain microbes in the mammalian gut are impacted by dietary iron levels and play important roles in host iron regulation. However, little is known currently about which specific microorganisms are most reliant on dietary iron and how the loss of these microbiome members may influence host health.

Here, we quantified the response of a complex, native microbiome in C57BL/6 mice to a subclinical period of iron deprivation. Our approach was unique compared to other studies with respect to carefully monitoring host-microbiome adaptation to a chemically defined diet (acclimatization) prior to a 2-week period of LI challenge. With this approach, we were able to partition significant changes in microbiome diversity due to dietary change versus iron removal, which turned out to be very important, as simply administering a new dietary regimen with or without normal iron levels potentially confounds these two influences. The murine host exhibited generally stable serum iron levels throughout LI challenge but showed markedly reduced stool iron excretion, which supports host systemic iron withholding. Consistent with these observations, several known iron-regulated transporters were upregulated in the small intestine of iron-deprived mice, yet they remained outwardly healthy with no signs of anemia.

Over the course of the same LI challenge, the microbiome underwent dramatic changes, and members of six common bacterial families were completely lost from >50% of the mice. We expect these species have the greatest dependence on dietary iron for survival, although it is possible that these observations were indirect effects, such as the loss or metabolic change of cross-feeding members in the community. Interestingly, all six of the most affected bacteria were anaerobes belonging to the most common phyla in the mammalian gut, *Firmicutes* (*Lachnospiraceae*, *Ruminococcaceae*) and *Bacteroidetes* (*Prevotellaceae*, *Porphyromonadaceae*, *Rikenellaceae*, and uncharacterized *Bacteroidales*). Moreover, three bacteria, each belonging to the *Bacteroidales* order, appeared to be hypersensitive to LI challenge, having gone below our detection limit over the relatively short (2-week) period of iron deprivation. This suggests that these *Bacteroidales* members are highly sensitive to low dietary iron.

Pathogenic species (e.g., *Pseudomonas*, *Staphylococcus*, and some *Enterobacteriaceae*) are often facultative anaerobes with robust, iron-utilizing metabolic capabilities that permit somewhat unchecked growth in host tissue (8). These capabilities include pathways for respiring a range of potentially available terminal electron acceptors; siderophores for competitively chelating and transporting Fe(III) through the cell wall/membrane; and enzymes for building and breaking down iron-protoporphyrin IX (heme) (9, 10). Metabolic versatility supports opportunistic infectious agents and/or bacteria that move through different environments as they travel between hosts. Iron acquisition is of particular importance, since iron is actively sequestered by the host during infection and since, especially as a constituent of heme, it is essential for the rapid growth that respiration enables. Consistent with iron-scavenging capabilities, facultative microbiome members did not appear to be as affected by the LI diet (i.e., not detected with OTU selection techniques), although mice in our vivarium and in this study did not carry high levels of these bacteria compared to the human gut microbiome. Additional studies are needed to adequately quantify the response of facultative anaerobes to dietary LI challenge.

In contrast to facultative anaerobes, iron metabolism in nonpathogenic members of the *Bacteroidetes* phylum is not well known. For example, despite characterization studies, these bacteria do not appear to possess complete metabolic pathways for synthesizing and degrading heme (11), even though it should be required, potentially as constituents of enzymes composing anaerobic respiratory pathways that they use preferentially *in lieu* of fermentative modes of growth (12). It is believed that these species obligately scavenge heme from the host to which they have adapted. The elimination of three *Bacteroidetes* OTUs (from *Prevotellaceae*, *Porphyromonadaceae*, and unclassified *Bacteroidales*) during LI challenge and their continued absence even after iron was reintroduced suggests that the cooperative link by which these anaerobes fill their iron requirement is irrevocably broken. This could have occurred if members of low-abundance bacterial species capable of directly supplying heme (e.g., Escherichia coli) also underwent extinction, keeping in mind that 16S sequencing, as we and others have shown, has significant detection bias for lower-abundance OTUs (13). Alternatively, if the host was the most important source of heme for these bacteria, they may have gone unfed during LI challenge as a result of robust host uptake and recycling. Regardless, the impact of LI challenge was akin to antibiotic treatment for these taxa. Mechanistic studies are now needed to better understand the direct impacts of LI challenge on bacterial extinction in the mammalian gut.

Nonpathogenic *Firmicutes* likewise frequently lack complete heme biosynthetic and degradative pathways (11). However, the heme requirement in these species is minimal, possibly because they are known to gain energy by nonrespiratory, fermentative means that do not depend on heme (12). Accordingly, the two *Lachnospiraceae* and *Ruminococcaceae* OTUs that were attenuated by LI challenge readily returned once iron was restored to the diet, suggesting the overall effect was not great enough to lead to extinction. However, why these OTUs and not others were impacted by LI challenge is not clear. As described above, it is possible that suppression was an indirect effect of community perturbation, but alternatively, these bacteria could have hitherto unappreciated iron/heme dependencies, uncovered by our approach. Further work, both *in vitro* and using mice populated by defined consortia containing defined representatives from this study, will clarify the roles of each species in collectively responding to changing iron supply.

The microbiome of different groups of mice varied somewhat in diversity at the outset of replicate experiments, which highlights the importance of replication in animal models that adequately account for litter-to-litter variability (14). Notably, our study encompassed both sexes and adults of different ages, but none of these variables impacted either the nature of the observed taxonomic shifts in microbiome diversity or its apparent irreversibility. The impact of an LI diet on the microbiome of experimental (laboratory-reared) mice has been examined previously in only a few studies (15–17). While all studies reported substantial shifts in taxonomic abundances, there was little similarity between them with respect to mouse strain (S129S6/SvEV [15] versus Swiss Webster [17] versus C57BL/6 [16]), acclimatization period following dietary shift (8 weeks [17] versus none [15, 16]), age at the start of the experiment (3 weeks old [16] versus 4 weeks old [17] versus 8 to 14 weeks old [15]), or the chemical form of iron used (ferric [17] versus ferrous [16] versus both ferric and ferrous iron [15]). More importantly, these studies differed markedly in the way the microbiome was characterized (bacterial culture [17] versus 16S rRNA V3-V4 sequencing [16] versus 16S rRNA V6 sequencing [15]). Our model/approach provides some standardization of these parameters. Other approaches described in rats (18) and pigs (19) also differed with respect to animal age/life stage (neonates [19] versus adolescents [18]) and the techniques used to evaluate the microbiome (quantitative PCR [qPCR] and PCR/temperature gradient gel electrophoresis [18] and Illumina-based 16S rRNA gene sequencing [19]), which again makes it somewhat impossible to compare LI impacts on specific microbiome taxonomic groups. It is important to note that the study described here was not conducted to compare the few reported low-iron challenge models. Rather, this study was designed to maximize reproducibility and inferences relevant to the microbial ecology of the mammalian gut. Regardless of the model, studies to date provide strong evidence that both iron depletion and repletion following LI challenge results in significant changes in mammalian gut microbiome diversity. Future studies focusing on iron metabolism of bacteria most susceptible to LI conditions will help clarify molecular mechanisms underlying these important changes and how microbial interactions with dietary iron ultimately influence host health and disease.

## MATERIALS AND METHODS

### Experimental animals and model overview.

In this study, all C57BL/6 mice were derived from breeders originally purchased from The Jackson Laboratory (Bar Harbor, ME). Mice were subsequently bred and maintained at the American Association for the Accreditation of Laboratory Animal Care-accredited Animal Resource Center (ARC) at Montana State University and housed under specific-pathogen-free conditions (including murine norovirus) in individually ventilated cages with sterilized bedding. Female and male mice were separated at weaning and housed separately until the start of each experiment. Mouse sex and age at the outset of each experiment is given in Table S1 in the supplemental material, and an overview of time points and experimental transitions is given in Fig. S1. Mice were reared on standard chow (diet 5013; Labdiet, St. Louis, MO) and switched to an iron-deficient but otherwise nutritionally sufficient chow (U8958 version 176; Scientific Animal Food and Engineering [SAFE], Augy, France) with iron supplementation as iron(II) sulfate heptahydrate (215422; Sigma-Aldrich, St. Louis, MO, USA) dissolved in distilled, purified water (Purelab water purification system; ELGA LabWater, Lane End, High Wycombe, UK). Water was made fresh in sterilized plastic bottles and changed every 3 days to account for the precipitation of iron. For low-iron challenge, dietary and living conditions were held constant, with the only difference being that iron-supplemented drinking water was replaced with regular vivarium drinking water (Hydropac AWS-2500 pouch machine; Lab Products, Seaford, DE, USA). Vivarium water is sourced using a reverse osmosis system, and iron levels were tested and confirmed to be less than the U.S. Environmental Protection Agency standard of 0.3 mg/liter or ppm. Longitudinal serum samples were prepared from blood sampled by the submandibular bleeding method (this level is well below residual iron levels in iron-depleted chow used for LI challenge). Stool samples were collected during the same visit as blood sampling. All animal experiments were approved by the Montana State University Institutional Animal Care and Use Committee. No statistical methods were used to select *a priori* sample sizes, and no randomization techniques or investigator blinding was included.

### Experimental groups.

Five different experiments were conducted to evaluate the effect of dietary iron on gut microbiome diversity, consisting of at least two technical replicate cages, as indicated in Table S1. The timeline of each experimental group refers to separate, nonoverlapping experiments conducted on different dates and indicative of biological replicates. Cages were composed of 5 mice each. Unfortunately, data were not available for all mice at each time point due to problems with DNA extraction, 16S rRNA amplification, and/or problems arising in the Illumina DNA sequencing pipeline.

### (i) Pilot group.

To establish methodology and efficacy of iron limitation, two cages of 9-week-old, female mice were switched from standard mouse chow to the low-iron chow and with iron sulfate in their drinking water as described above. After 2 weeks (*n* = 14 days), the water was replaced with metal-free purified water for another 2 weeks. Stool samples were collected throughout the experiment.

### (ii) Validation groups 1 and 2.

To evaluate iron limitation on host expression of iron metabolic genes, two experimental groups were designed to have specific endpoints as indicated. Blood and stool were sampled throughout the experiment as indicated, and half of the mice in group 3 (*n* = 5) were transitioned to iron-replete water for the 2 weeks following the iron challenge. Duodenal and cecal tissues were harvested following euthanasia for evaluation of host iron responses.

### (iii) Repletion groups 1 and 2.

Two additional groups were used to increase biological replication and to consider a group of male mice (repletion group 2). Both groups were treated identically to validation group 2, with the only experimental difference being that no samples were considered at the outset of the experiments (*T*_0_).

### Quantification of iron in serum and stool.

Weekly blood samples (<60 μl) were collected and frozen (–20°C) until prepared for total iron quantification using inductively coupled plasma-mass spectrometry (ICP-MS). Blood was thawed at room temperature and centrifuged for 10 min at 1,677 × *g* to separate the serum. A known volume (∼20 μl) of serum was removed from the top of each sample and incubated in 1 ml of 50% nitric acid (nitric acid optima and trace metal grades; Fisher Scientific, Hampton, NH, USA) at 55°C for 30 to 60 min or until dissolved. Samples were diluted with 9 ml of metal-free water (Purelab water purification system; ELGA LabWater, High Wycombe, UK) and centrifuged at 5,700 × *g* for 10 min. After centrifugation, samples were filtered to remove particulates (0.2-μm syringe filters; Corning Inc., Corning, NY, USA). Stool pellets were dried overnight under vacuum and weighed before being incubated with 1 ml 50% nitric acid at 55°C for 1 h. Samples were diluted with 9 ml of metal-free water and centrifuged at 5,700 × *g* for 10 min before filtration to remove particulates. All samples were stored in the dark at room temperature until analysis by ICP-MS. Total iron in serum and stool was quantified using an Agilent 7500ce ICP-MS with a collision cell and a certified environmental calibration standard (product no. 4400-12 1116NCO2; CPI International, Santa Rosa, CA, USA). Total iron per sample was calculated based on volume (serum) or dry mass (stool).

### Transcriptional analysis of iron-responsive mouse genes.

Duodenal and cecal tissues were immediately harvested from euthanized mice, snap-frozen in liquid nitrogen, and stored at −80°C. For processing, tissue was thawed on ice and lysed by manual grinding using pestles, followed by water bath sonication (program: pulse of 10 s on, 10 s off, 80% amplitude for 2 min total; Fisherbrand model 505 sonic dismembrator; Fisher Scientific, Hampton, NH, USA) and shredding (QIAshredder, Qiagen, Hilden, Germany). Total RNA was extracted using a kit (RNeasy minikit; Qiagen, Hilden, Germany), quantified using a spectrophotometer (ND-1000; NanoDrop), and stored at –80°C until further use. cDNA was prepared using iScript polymerase master mix (Bio-Rad, Hercules, CA, USA) and thermocycling (program: 25°C for 5 min, 46°C for 20 min, 95°C for 1 min, 4°C hold; Applied Biosciences model 2720; Beverly Hills, CA, USA). Quantitative PCR was performed on a Roche 96 light cycler using a SYBR green fluorescence assay (AzuraQuant green fast qPCR mix; Azura Genomics, Raynham, MA, USA). Transcription of genes encoding duodenal cytochrome *b* (Dcytb), divalent metal transporter 1 (Dmt1), and ferroportin were evaluated relative to β-actin using primer sequences and sources listed in Table 1. Primers were synthesized (Integrated DNA Technologies, Coralville, IA, USA) and diluted to a working concentration of 10 μM. qPCR conditions were denaturation at 95°C for 10 min, followed by 40 cycles of 95°C for 15 s and 60°C for 60 s. Cycle thresholds were estimated from technical replicates and used to analyze relative gene expression using the comparative threshold cycle (*C_T_*) method (20).

**TABLE 1:** Primer sequences for transcriptional analysis of iron-responsive mouse genes

Gene product	Forward (5′–3′)	Reverse (5′–3′)	Source
Dcytb	GCAGCGGGCTCGAGTTTA	TTCCAGGTCCATGGCAGTCT	Dupic et al. (32)
Dmt1	CGGAGTCCTCATCACCATCG	CTGGCTGGGCTTCACTGTAA	NCBI primer design tool
Ferroportin	TTGCAGGAGTCATTGCTGCTA	TGGAGTTCTGCACACCATTGAT	Dupic et al. (32)
β-Actin	AACCCTAAGGCCAACCGTGAA	TCACGCACGATTTCCCTCTCA	NCBI primer design tool

### Bacterial 16S rRNA sequencing and processing.

DNA was extracted using a DNeasy Powersoil kit (Qiagen, Hilden, Germany) from stool pellets by following the manufacturer’s instructions. DNA was shipped in 96-well plates to the University of Michigan Center for Microbial Systems for Illumina paired-end (2 × 250 bp) sequencing of the variable region 4 (V4) of the bacterial 16S rRNA gene. Raw sequencing reads were processed using mothur v.1.39.5. Low-quality reads were removed by following the mothur standard operating procedure (21) (accessed on 7 May 2017), and forward and reverse reads were assembled into contigs. Contigs containing ambiguous bases or homopolymers of >8 bp were removed. Identical sequences were combined and aligned against the V4 region of the 16S rRNA alignment of the SILVA database (version 128). Chimeric sequences were identified with UCHIME (22) and removed. Operational taxonomic units (OTUs) were defined as sharing ≥97% sequence identity and were binned using VSEARCH (23). OTUs represented by fewer than 100 reads in the entire data set were removed to guard against spurious observations. The remaining OTUs were taxonomically classified within mothur using the Ribosomal Database Project’s Bayesian classifier (training set 10) (24), and all OTUs that classified to mitochondria, chloroplasts, or *Eukaryota* or that remained unclassified at the domain level were removed. For normalization, each sample was rarefied to 10,000 reads.

### Statistical methods.

Overall microbiome diversity was estimated using the inverse Simpson index (α-diversity), and ordinations (nonmetric multidimensional scaling [NMDS]) were performed to visualize patterns of microbiome similarity (β-diversity). Both analyses are based on the presence-absence and relative abundance of taxa (OTUs) resolved by 16S sequencing, but where α-diversity estimates represent diversity within a single community, β-diversity represents diversity between several different communities. These analyses as well as bar charts representing taxon abundance were generated in R version 3.6.0 (25) using vegan 2.5–3 (26), labdsv 1.8 (27), and custom scripts. Longitudinal data were analyzed in R using paired, parametric *t* test for normal data and nonparametric Wilcoxon rank sum test for nonnormal data. Changes in microbiome diversity between experimental treatments were tested for significance using PERMANOVA (adonis2 in vegan). OTUs most responsible for these changes were identified as described in Results using indicator value (IndVal [28, 29] in labdsv) and random forest (30) analyses or by multiple *t* testing with *P* value correction for multiple comparisons (Benjamini and Hochberg method [31] in R).

### Data availability.

Sequencing reads were deposited in the National Center for Biotechnology Information (NCBI) BioProject database with accession number PRJNA562734.

## Supplementary Material

Supplemental file 1

## References

[1] Arosio P, Levi S 2010 Cytosolic and mitochondrial ferritins in the regulation of cellular iron homeostasis and oxidative damage. Biochim Biophys Acta 1800:783–792. doi:10.1016/j.bbagen.2010.02.005. 20176086

[2] Andrews NC, Schmidt PJ 2007 Iron homeostasis. Annu Rev Physiol 69:69–85. doi:10.1146/annurev.physiol.69.031905.164337. 17014365

[3] Schop A, Stouten K, Riedl JA, van Houten RJ, Leening MJG, van Rosmalen J, Bindels PJE, Levin MD 2020 A new diagnostic work-up for defining anemia etiologies: a cohort study in patients ≥ 50 years in general practices. BMC Fam Pract 21:167. doi:10.1186/s12875-020-01241-7. 32799818 PMC7429725

[4] Hurrell R, Egli I 2010 Iron bioavailability and dietary reference values. Am J Clin Nutr 91:1461S–1467S. doi:10.3945/ajcn.2010.28674F. 20200263

[5] N I, Derrien M, van Doorn GM, Rijnierse A, van den Bogert B, Muller M, Dekker J, Kleerebezem M, van der Meer R 2012 Dietary heme alters microbiota and mucosa of mouse colon without functional changes in host-microbe cross-talk. PLoS One 7:e49868. doi:10.1371/journal.pone.0049868. 23239972 PMC3519815

[6] Zimmer J, Lange B, Frick JS, Sauer H, Zimmermann K, Schwiertz A, Rusch K, Klosterhalfen S, Enck P 2012 A vegan or vegetarian diet substantially alters the human colonic faecal microbiota. Eur J Clin Nutr 66:53–60. doi:10.1038/ejcn.2011.141. 21811294

[7] Das NK, Schwartz AJ, Barthel G, Inohara N, Liu Q, Sankar A, Hill DR, Ma X, Lamberg O, Schnizlein MK, Arques JL, Spence JR, Nunez G, Patterson AD, Sun D, Young VB, Shah YM 2020 Microbial metabolite signaling is required for systemic iron homeostasis. Cell Metab 31:115–130. doi:10.1016/j.cmet.2019.10.005. 31708445 PMC6949377

[8] Hammer ND, Skaar EP 2011 Molecular mechanisms of Staphylococcus aureus iron acquisition. Annu Rev Microbiol 65:129–147. doi:10.1146/annurev-micro-090110-102851. 21639791 PMC3807827

[9] Richard KL, Kelley BR, Johnson JG 2019 Heme uptake and utilization by gram-negative bacterial pathogens. Front Cell Infect Microbiol 9:81. doi:10.3389/fcimb.2019.00081. 30984629 PMC6449446

[10] Sheldon JR, Heinrichs DE 2015 Recent developments in understanding the iron acquisition strategies of gram positive pathogens. FEMS Microbiol Rev 39:592–630. doi:10.1093/femsre/fuv009. 25862688

[11] Dailey HA, Dailey TA, Gerdes S, Jahn D, Jahn M, O'Brian MR, Warren MJ 2017 Prokaryotic heme biosynthesis: multiple pathways to a common essential product. Microbiol Mol Biol Rev 81:e00048-16. doi:10.1128/MMBR.00048-16. 28123057 PMC5312243

[12] Gruss A, Borezee-Durant E, Lechardeur D 2012 Environmental heme utilization by heme-auxotrophic bacteria. Adv Microb Physiol 61:69–124. doi:10.1016/B978-0-12-394423-8.00003-2. 23046952

[13] Martinson JNV, Pinkham NV, Peters GW, Cho H, Heng J, Rauch M, Broadaway SC, Walk ST 2019 Rethinking gut microbiome residency and the Enterobacteriaceae in healthy human adults. ISME J 13:2306–2318. doi:10.1038/s41396-019-0435-7. 31089259 PMC6776003

[14] Katsnelson A 2019 Minding the microbiome of your mice. Lab Anim 48:313–315. doi:10.1038/s41684-019-0424-5. 31645694

[15] Ellermann M, Gharaibeh RZ, Maharshak N, Perez-Chanona E, Jobin C, Carroll IM, Arthur JC, Plevy SE, Fodor AA, Brouwer CR, Sartor RB 2020 Dietary iron variably modulates assembly of the intestinal microbiota in colitis-resistant and colitis-susceptible mice. Gut Microbes 11:32–50. doi:10.1080/19490976.2019.1599794. 31179826 PMC6973310

[16] La Carpia F, Wojczyk BS, Annavajhala MK, Rebbaa A, Culp-Hill R, D'Alessandro A, Freedberg DE, Uhlemann AC, Hod EA 2019 Transfusional iron overload and intravenous iron infusions modify the mouse gut microbiota similarly to dietary iron. NPJ Biofilms Microbiomes 5:26. doi:10.1038/s41522-019-0097-2. 31583109 PMC6760189

[17] Tompkins GR, O'Dell NL, Bryson IT, Pennington CB 2001 The effects of dietary ferric iron and iron deprivation on the bacterial composition of the mouse intestine. Curr Microbiol 43:38–42. doi:10.1007/s002840010257. 11375662

[18] Dostal A, Chassard C, Hilty FM, Zimmermann MB, Jaeggi T, Rossi S, Lacroix C 2012 Iron depletion and repletion with ferrous sulfate or electrolytic iron modifies the composition and metabolic activity of the gut microbiota in rats. J Nutr 142:271–277. doi:10.3945/jn.111.148643. 22190022 PMC3260059

[19] Knight LC, Wang M, Donovan SM, Dilger RN 2019 Early-life iron deficiency and subsequent repletion alters development of the colonic microbiota in the pig. Front Nutr 6:120. doi:10.3389/fnut.2019.00120. 31440513 PMC6692694

[20] Schmittgen TD, Livak KJ 2008 Analyzing real-time PCR data by the comparative *C*_T_ method. Nat Protoc 3:1101–1108. doi:10.1038/nprot.2008.73. 18546601

[21] Kozich JJ, Westcott SL, Baxter NT, Highlander SK, Schloss PD 2013 Development of a dual-index sequencing strategy and curation pipeline for analyzing amplicon sequence data on the MiSeq Illumina sequencing platform. Appl Environ Microbiol 79:5112–5120. doi:10.1128/AEM.01043-13. 23793624 PMC3753973

[22] Edgar RC, Haas BJ, Clemente JC, Quince C, Knight R 2011 UCHIME improves sensitivity and speed of chimera detection. Bioinformatics 27:2194–2200. doi:10.1093/bioinformatics/btr381. 21700674 PMC3150044

[23] Rognes T, Flouri T, Nichols B, Quince C, Mahe F 2016 VSEARCH: a versatile open source tool for metagenomics. PeerJ 4:e2584. doi:10.7717/peerj.2584. 27781170 PMC5075697

[24] Cole JR, Wang Q, Fish JA, Chai B, McGarrell DM, Sun Y, Brown CT, Porras-Alfaro A, Kuske CR, Tiedje JM 2014 Ribosomal Database Project: data and tools for high throughput rRNA analysis. Nucleic Acids Res 42:D633–D642. doi:10.1093/nar/gkt1244. 24288368 PMC3965039

[25] R Core Development Team. 2018 R: a language and environment for statistical computing, R Foundation for Statistical Computing, Vienna, Austria https://www.R-project.org/.

[26] Oksanen J, Guillaume Blanchet F, Friendly M, Kindt R, Legendre P, McGlinn D, Minchin PR, O'Hara RB, Simpson GL, Solymos P, Stevens MH, Szoecs E, Wagner H 2018 vegan: community ecology package. https://cran.r-project.org/web/packages/vegan/index.html.

[27] Roberts DW 2019 labdsv: ordination and multivariate analysis for ecology, v2.0–1. https://cran.r-project.org/web/packages/labdsv/index.html.

[28] De Caceres M, Legendre P 2009 Associations between species and groups of sites: indices and statistical inference. Ecology 90:3566–3574. doi:10.1890/08-1823.1. 20120823

[29] Dufrene M, Legendre P 1997 Species assemblages and indicator species: the need for a flexible asymmetrical approach. Ecol Monogr 67:345–366. doi:10.2307/2963459.

[30] Liaw A, Wiener M 2002 Classification and regression by randomForest. R News 2:18–22.

[31] Benjamini Y, Hochberg Y 1995 Controlling the false discovery rate: a practical and powerful approach to multiple testing. J R Stat Soc Series B Stat Methodol 57:289–300. doi:10.1111/j.2517-6161.1995.tb02031.x.

[32] Dupic F, Fruchon S, Bensaid M, Loreal O, Brissot P, Borot N, Roth MP, Coppin H 2002 Duodenal mRNA expression of iron related genes in response to iron loading and iron deficiency in four strains of mice. Gut 51:648–653. doi:10.1136/gut.51.5.648. 12377801 PMC1773425

