# Supplementary material for: Dynamic Gut Microbiome Changes in Response to Low-Iron Challenge
Source: Appl Environ Microbiol. 2021 Jan 15;87(3):e02307-20. doi: 10.1128/AEM.02307-20 (PMC7848906; doi:10.1128/AEM.02307-20)
Supplement: Supplemental file 1 [file AEM.02307-20-s0001.pdf]

Supplementary Table 1. Sex, age, and number of mice used in each experimental group. In Validation Group 1 and Repletion Groups 1 and 2, half of the mice were evaluated at the end of low-iron (LI) challenge and the other half at the end of the repletion phase.

Group	Sex	Age at T_0 (weeks)	Total # of mice	Treatment/Transitions	# of mice in treatment/transition
Pilot	F	9	10	$T_0 \rightarrow B_{14} \rightarrow LI_7 \rightarrow LI_{14}$	10
Validation 1	F	11	10	$T_0 \rightarrow B_7 \rightarrow B_{14} \rightarrow LI_7 \rightarrow LI_{14}$	10
Validation 2	F	11	10	$T_0 \rightarrow B_7 \rightarrow B_{14} \rightarrow LI_7 \rightarrow LI_{14}$	5
				$T_0 \rightarrow B_7 \rightarrow B_{14} \rightarrow LI_7 \rightarrow LI_{14} \rightarrow R_7 \rightarrow R_{14}$	5
Repletion 1	F	10	10	$B_7 \rightarrow B_{14} \rightarrow LI_7 \rightarrow LI_{14}$	5
				$B_7 \rightarrow B_{14} \rightarrow LI_7 \rightarrow LI_{14} \rightarrow R_7 \rightarrow R_{14}$	5
Repletion 2	M	15	10	$B_7 \rightarrow B_{14} \rightarrow LI_7 \rightarrow LI_{14}$	5
				$B_7 \rightarrow B_{14} \rightarrow LI_7 \rightarrow LI_{14} \rightarrow R_7 \rightarrow R_{14}$	5

Supplementary Figure 1. Overview of timepoints and experimental transitions for groups shown in Supplementary Table 1.

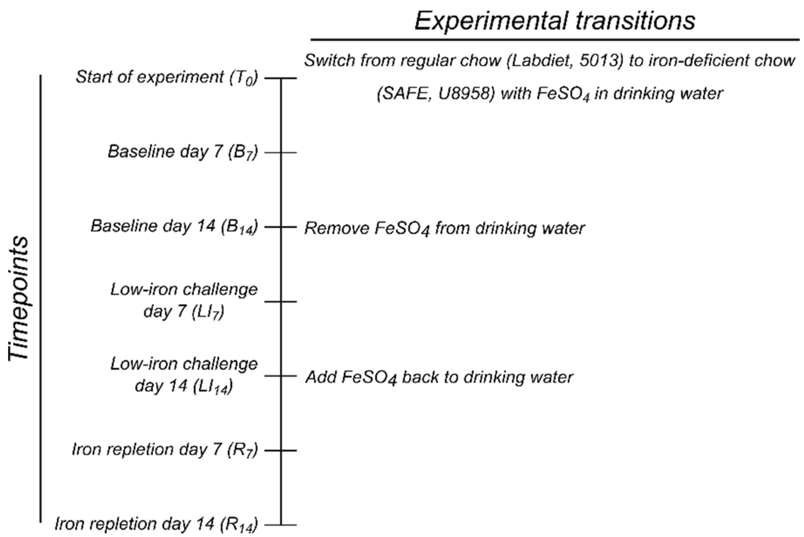

Supplementary Figure 2. Median alpha (top) and beta (bottom) diversity across all experiments. Beta diversity represents community dissimilarity to mice in all other groups. Estimates at Baseline (B), Low-Iron (LI), and Repletion (R) represent combined timepoints (day 7 and 14) if available (see Supplementary Table 1).

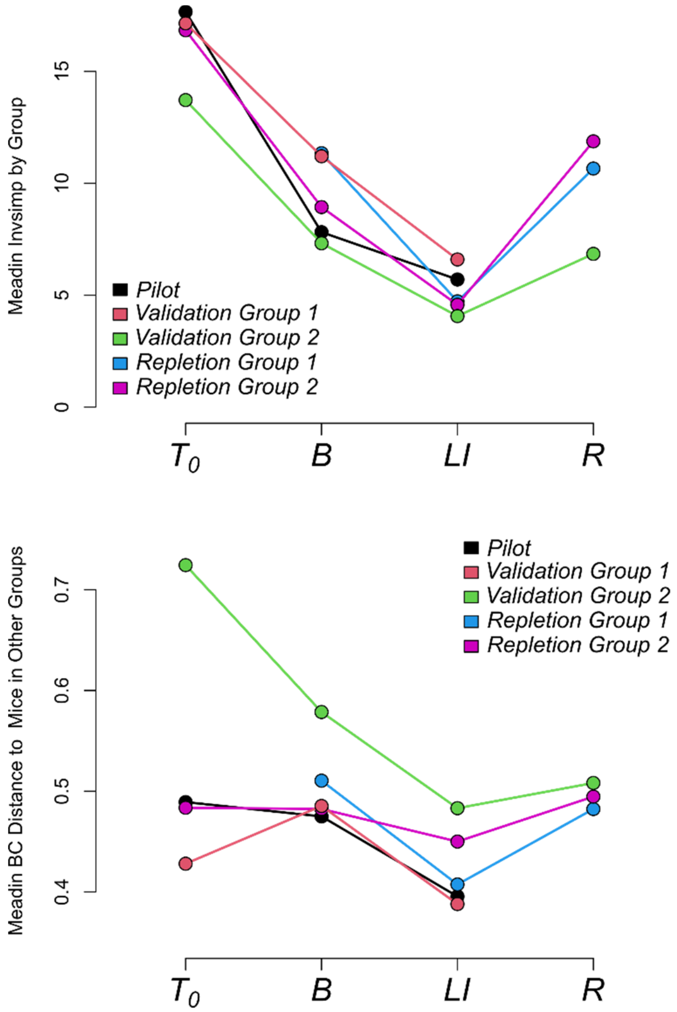

Supplementary Table 2. Reduction in PERMANOVA pseudo-F statistic by OTU selection method across experimental treatment transitions and experimental group. RF = random forest, INDVAL = indicator value analysis, P-A = OTU presence-absence, T-TEST = t-test of OTU abundance

TRANSITION	METHOD	PILOT GROUP	VALIDATION GROUP 1	VALIDATION GROUP 2	REPLETION GROUP 1	REPLETION GROUP 2
T_0 to B_7	RF	NA	0.831	0.756	NA	NA
	INDVAL	NA	0.871	0.401	NA	NA
	P-A	NA	-0.003	-0.007	NA	NA
	T-TEST	NA	0.664	0.857	NA	NA
B_{14} to LI_7	RF	0.792	0.641	0.844	0.522	NA
	INDVAL	0.666	0.632	0.610	0.495	NA
	P-A	0.474	0.314	0.125	0.183	NA
	T-TEST	0.084	0.363	0.595	0.134	NA
LI_{14} to R_7	RF	NA	NA	0.822	0.345	0.591
	INDVAL	NA	NA	0.892	0.227	0.558
	P-A	NA	NA	0.003	-0.028	0.084
	T-TEST	NA	NA	0.800	0.263	0.393

Supplementary Table 3. Number of OTUs selected by selection method across experimental treatment transitions and experimental group. RF = random forest, INDVAL = indicator value analysis, P-A = OTU presence-absence, T-TEST = t-test of OTU abundance

TRANSITION	METHOD	PILOT GROUP	VALIDATION GROUP 1	VALIDATION GROUP 2	REPLETION GROUP 1	REPLETION GROUP 2
T_0 to B_7	RF	NA	52	24	NA	NA
	INDVAL	NA	69	34	NA	NA
	P-A	NA	67	31	NA	NA
	T-TEST	NA	76	60	NA	NA
B_{14} to LI_7	RF	32	40	29	40	NA
	INDVAL	49	63	37	69	NA
	P-A	95	78	61	95	NA
	T-TEST	32	47	31	47	NA
LI_{14} to R_7	RF	NA	NA	19	18	20
	INDVAL	NA	NA	25	19	20
	P-A	NA	NA	56	53	56
	T-TEST	NA	NA	15	14	17

REFERENCES

1 Dupic, F. *et al.* Duodenal mRNA expression of iron related genes in response to iron loading and iron deficiency in four strains of mice. *Gut* **51**, 648-653, doi:10.1136/gut.51.5.648 (2002).

TRANSITION	GROUP	CHANGE	OTU	FAMILY
T0 TO B7	VALIDATION 1	INCREASE	Otu0003	Erysipelotrichaceae
T0 TO B7	VALIDATION 1	INCREASE	Otu0005	Porphyromonadaceae
T0 TO B7	VALIDATION 1	INCREASE	Otu0006	Porphyromonadaceae
T0 TO B7	VALIDATION 1	INCREASE	Otu0007	Bacteroidaceae
T0 TO B7	VALIDATION 1	INCREASE	Otu0009	Prevotellaceae
T0 TO B7	VALIDATION 1	INCREASE	Otu0010	Erysipelotrichaceae
T0 TO B7	VALIDATION 1	INCREASE	Otu0011	Lachnospiraceae
T0 TO B7	VALIDATION 1	INCREASE	Otu0019	Porphyromonadaceae
T0 TO B7	VALIDATION 1	INCREASE	Otu0049	Porphyromonadaceae
T0 TO B7	VALIDATION 1	INCREASE	Otu0099	Porphyromonadaceae
T0 TO B7	VALIDATION 1	INCREASE	Otu0183	Coriobacteriaceae
T0 TO B7	VALIDATION 1	DECREASE	Otu0014	Prevotellaceae
T0 TO B7	VALIDATION 1	DECREASE	Otu0015	Lachnospiraceae
T0 TO B7	VALIDATION 1	DECREASE	Otu0021	Rikenellaceae
T0 TO B7	VALIDATION 1	DECREASE	Otu0022	Porphyromonadaceae
T0 TO B7	VALIDATION 1	DECREASE	Otu0030	Porphyromonadaceae
T0 TO B7	VALIDATION 1	DECREASE	Otu0031	Porphyromonadaceae
T0 TO B7	VALIDATION 1	DECREASE	Otu0039	Porphyromonadaceae
T0 TO B7	VALIDATION 1	DECREASE	Otu0041	Porphyromonadaceae
T0 TO B7	VALIDATION 1	DECREASE	Otu0051	Porphyromonadaceae
T0 TO B7	VALIDATION 1	DECREASE	Otu0052	Porphyromonadaceae
T0 TO B7	VALIDATION 1	DECREASE	Otu0056	Rikenellaceae
T0 TO B7	VALIDATION 1	DECREASE	Otu0072	Porphyromonadaceae
T0 TO B7	VALIDATION 1	DECREASE	Otu0080	Clostridiaceae_1
T0 TO B7	VALIDATION 1	DECREASE	Otu0081	Rikenellaceae
T0 TO B7	VALIDATION 1	DECREASE	Otu0084	Porphyromonadaceae
T0 TO B7	VALIDATION 1	DECREASE	Otu0095	Lachnospiraceae
T0 TO B7	VALIDATION 1	DECREASE	Otu0096	Porphyromonadaceae
T0 TO B7	VALIDATION 1	DECREASE	Otu0107	Anaeroplasmataceae
T0 TO B7	VALIDATION 1	DECREASE	Otu0118	Porphyromonadaceae
T0 TO B7	VALIDATION 1	DECREASE	Otu0124	Porphyromonadaceae
T0 TO B7	VALIDATION 1	DECREASE	Otu0127	Ruminococcaceae
T0 TO B7	VALIDATION 1	DECREASE	Otu0129	Lactobacillaceae
T0 TO B7	VALIDATION 1	DECREASE	Otu0131	Ruminococcaceae
T0 TO B7	VALIDATION 1	DECREASE	Otu0132	Lachnospiraceae
T0 TO B7	VALIDATION 1	DECREASE	Otu0135	Deltaproteobacteria_unclassified
T0 TO B7	VALIDATION 1	DECREASE	Otu0140	Porphyromonadaceae
T0 TO B7	VALIDATION 1	DECREASE	Otu0142	Firmicutes_unclassified
T0 TO B7	VALIDATION 1	DECREASE	Otu0143	Porphyromonadaceae
T0 TO B7	VALIDATION 1	DECREASE	Otu0146	Bacteroidales_unclassified
T0 TO B7	VALIDATION 1	DECREASE	Otu0148	Lachnospiraceae
T0 TO B7	VALIDATION 1	DECREASE	Otu0151	Porphyromonadaceae
T0 TO B7	VALIDATION 1	DECREASE	Otu0168	Clostridiales_unclassified
T0 TO B7	VALIDATION 1	DECREASE	Otu0171	Ruminococcaceae
T0 TO B7	VALIDATION 1	DECREASE	Otu0186	Clostridiales_unclassified
T0 TO B7	VALIDATION 1	DECREASE	Otu0196	Lachnospiraceae

T0 TO B7	VALIDATION	1	DECREASE	Otu0199	Ruminococcaceae
T0 TO B7	VALIDATION	1	DECREASE	Otu0234	Clostridia_unclassified
T0 TO B7	VALIDATION	1	DECREASE	Otu0239	Firmicutes_unclassified
T0 TO B7	VALIDATION	1	DECREASE	Otu0245	Lachnospiraceae
T0 TO B7	VALIDATION	1	DECREASE	Otu0304	Clostridiales_unclassified
T0 TO B7	VALIDATION	1	DECREASE	Otu0325	Clostridiales_unclassified
T0 TO B7	VALIDATION	2	INCREASE	Otu0001	Verrucomicrobiaceae
T0 TO B7	VALIDATION	2	INCREASE	Otu0010	Erysipelotrichaceae
T0 TO B7	VALIDATION	2	INCREASE	Otu0011	Lachnospiraceae
T0 TO B7	VALIDATION	2	INCREASE	Otu0012	Lachnospiraceae
T0 TO B7	VALIDATION	2	INCREASE	Otu0017	Erysipelotrichaceae
T0 TO B7	VALIDATION	2	INCREASE	Otu0024	Lachnospiraceae
T0 TO B7	VALIDATION	2	INCREASE	Otu0035	Lachnospiraceae
T0 TO B7	VALIDATION	2	INCREASE	Otu0045	Lachnospiraceae
T0 TO B7	VALIDATION	2	INCREASE	Otu0065	Lachnospiraceae
T0 TO B7	VALIDATION	2	INCREASE	Otu0136	Lachnospiraceae
T0 TO B7	VALIDATION	2	DECREASE	Otu0002	Bacteroidaceae
T0 TO B7	VALIDATION	2	DECREASE	Otu0021	Rikenellaceae
T0 TO B7	VALIDATION	2	DECREASE	Otu0029	Lactobacillaceae
T0 TO B7	VALIDATION	2	DECREASE	Otu0052	Porphyromonadaceae
T0 TO B7	VALIDATION	2	DECREASE	Otu0077	Erysipelotrichaceae
T0 TO B7	VALIDATION	2	DECREASE	Otu0087	Lachnospiraceae
T0 TO B7	VALIDATION	2	DECREASE	Otu0133	Lachnospiraceae
T0 TO B7	VALIDATION	2	DECREASE	Otu0139	Lachnospiraceae
T0 TO B7	VALIDATION	2	DECREASE	Otu0149	Lachnospiraceae
T0 TO B7	VALIDATION	2	DECREASE	Otu0201	Lachnospiraceae
T0 TO B7	VALIDATION	2	DECREASE	Otu0221	Lachnospiraceae
T0 TO B7	VALIDATION	2	DECREASE	Otu0272	Bacteria_unclassified
T0 TO B7	VALIDATION	2	DECREASE	Otu0303	Lachnospiraceae
T0 TO B7	VALIDATION	2	DECREASE	Otu0318	Bacteria_unclassified
B7 TO B14	VALIDATION	1	INCREASE	Otu0008	Clostridiales_unclassified
B7 TO B14	VALIDATION	1	INCREASE	Otu0020	Lachnospiraceae
B7 TO B14	VALIDATION	1	INCREASE	Otu0025	Porphyromonadaceae
B7 TO B14	VALIDATION	1	INCREASE	Otu0065	Lachnospiraceae
B7 TO B14	VALIDATION	1	INCREASE	Otu0067	Lachnospiraceae
B7 TO B14	VALIDATION	1	INCREASE	Otu0069	Lachnospiraceae
B7 TO B14	VALIDATION	1	INCREASE	Otu0142	Firmicutes_unclassified
B7 TO B14	VALIDATION	1	INCREASE	Otu0166	Lachnospiraceae
B7 TO B14	VALIDATION	1	INCREASE	Otu0169	Firmicutes_unclassified
B7 TO B14	VALIDATION	1	INCREASE	Otu0241	Bacteria_unclassified
B7 TO B14	VALIDATION	1	DECREASE	Otu0022	Porphyromonadaceae
B7 TO B14	VALIDATION	1	DECREASE	Otu0029	Lactobacillaceae
B7 TO B14	VALIDATION	1	DECREASE	Otu0031	Porphyromonadaceae
B7 TO B14	VALIDATION	1	DECREASE	Otu0033	Erysipelotrichaceae
B7 TO B14	VALIDATION	1	DECREASE	Otu0049	Porphyromonadaceae
B7 TO B14	VALIDATION	1	DECREASE	Otu0055	Lachnospiraceae
B7 TO B14	VALIDATION	1	DECREASE	Otu0070	Coriobacteriaceae

B7 TO B14	VALIDATION 1	DECREASE	Otu0084	Porphyromonadaceae
B7 TO B14	VALIDATION 1	DECREASE	Otu0113	Lachnospiraceae
B7 TO B14	VALIDATION 1	DECREASE	Otu0224	Lachnospiraceae
B7 TO B14	VALIDATION 2	INCREASE	Otu0001	Verrucomicrobiaceae
B7 TO B14	VALIDATION 2	INCREASE	Otu0010	Erysipelotrichaceae
B7 TO B14	VALIDATION 2	INCREASE	Otu0045	Lachnospiraceae
B7 TO B14	VALIDATION 2	INCREASE	Otu0076	Lachnospiraceae
B7 TO B14	VALIDATION 2	INCREASE	Otu0122	Lachnospiraceae
B7 TO B14	VALIDATION 2	INCREASE	Otu0159	Ruminococcaceae
B7 TO B14	VALIDATION 2	INCREASE	Otu0184	Bacteria_unclassified
B7 TO B14	VALIDATION 2	DECREASE	Otu0004	Bifidobacteriaceae
B7 TO B14	VALIDATION 2	DECREASE	Otu0017	Erysipelotrichaceae
B7 TO B14	VALIDATION 2	DECREASE	Otu0022	Porphyromonadaceae
B7 TO B14	VALIDATION 2	DECREASE	Otu0029	Lactobacillaceae
B7 TO B14	VALIDATION 2	DECREASE	Otu0044	Erysipelotrichaceae
B7 TO B14	VALIDATION 2	DECREASE	Otu0080	Clostridiaceae_1
B7 TO B14	VALIDATION 2	DECREASE	Otu0085	Ruminococcaceae
B7 TO B14	VALIDATION 2	DECREASE	Otu0100	Lachnospiraceae
B7 TO B14	VALIDATION 2	DECREASE	Otu0128	Lachnospiraceae
B7 TO B14	VALIDATION 2	DECREASE	Otu0175	Lachnospiraceae
B7 TO B14	REPLETION 1	INCREASE	Otu0011	Lachnospiraceae
B7 TO B14	REPLETION 1	INCREASE	Otu0012	Lachnospiraceae
B7 TO B14	REPLETION 1	INCREASE	Otu0013	Lachnospiraceae
B7 TO B14	REPLETION 1	INCREASE	Otu0035	Lachnospiraceae
B7 TO B14	REPLETION 1	INCREASE	Otu0036	Lachnospiraceae
B7 TO B14	REPLETION 1	INCREASE	Otu0045	Lachnospiraceae
B7 TO B14	REPLETION 1	INCREASE	Otu0047	Lachnospiraceae
B7 TO B14	REPLETION 1	INCREASE	Otu0053	Ruminococcaceae
B7 TO B14	REPLETION 1	INCREASE	Otu0069	Lachnospiraceae
B7 TO B14	REPLETION 1	INCREASE	Otu0075	Lachnospiraceae
B7 TO B14	REPLETION 1	INCREASE	Otu0106	Lachnospiraceae
B7 TO B14	REPLETION 1	INCREASE	Otu0111	Lachnospiraceae
B7 TO B14	REPLETION 1	INCREASE	Otu0130	Lachnospiraceae
B7 TO B14	REPLETION 1	INCREASE	Otu0158	Ruminococcaceae
B7 TO B14	REPLETION 1	INCREASE	Otu0188	Lachnospiraceae
B7 TO B14	REPLETION 1	INCREASE	Otu0213	Ruminococcaceae
B7 TO B14	REPLETION 1	INCREASE	Otu0311	Lachnospiraceae
B7 TO B14	REPLETION 1	DECREASE	Otu0001	Verrucomicrobiaceae
B7 TO B14	REPLETION 1	DECREASE	Otu0010	Erysipelotrichaceae
B7 TO B14	REPLETION 1	DECREASE	Otu0014	Prevotellaceae
B7 TO B14	REPLETION 1	DECREASE	Otu0022	Porphyromonadaceae
B7 TO B14	REPLETION 1	DECREASE	Otu0027	Ruminococcaceae
B7 TO B14	REPLETION 1	DECREASE	Otu0116	Porphyromonadaceae
B7 TO B14	REPLETION 1	DECREASE	Otu0170	Ruminococcaceae
B7 TO B14	REPLETION 1	DECREASE	Otu0204	Ruminococcaceae
B14 TO LI7	PILOT	INCREASE	Otu0001	Verrucomicrobiaceae
B14 TO LI7	PILOT	INCREASE	Otu0004	Bifidobacteriaceae

B14 TO LI7	PILOT	INCREASE	Otu0005	Porphyromonadaceae
B14 TO LI7	PILOT	INCREASE	Otu0020	Lachnospiraceae
B14 TO LI7	PILOT	INCREASE	Otu0031	Porphyromonadaceae
B14 TO LI7	PILOT	DECREASE	Otu0009	Prevotellaceae
B14 TO LI7	PILOT	DECREASE	Otu0014	Prevotellaceae
B14 TO LI7	PILOT	DECREASE	Otu0015	Lachnospiraceae
B14 TO LI7	PILOT	DECREASE	Otu0019	Porphyromonadaceae
B14 TO LI7	PILOT	DECREASE	Otu0021	Rikenellaceae
B14 TO LI7	PILOT	DECREASE	Otu0032	Bacteroidales_unclassified
B14 TO LI7	PILOT	DECREASE	Otu0033	Erysipelotrichaceae
B14 TO LI7	PILOT	DECREASE	Otu0041	Porphyromonadaceae
B14 TO LI7	PILOT	DECREASE	Otu0046	Porphyromonadaceae
B14 TO LI7	PILOT	DECREASE	Otu0048	Clostridiales_unclassified
B14 TO LI7	PILOT	DECREASE	Otu0049	Porphyromonadaceae
B14 TO LI7	PILOT	DECREASE	Otu0052	Porphyromonadaceae
B14 TO LI7	PILOT	DECREASE	Otu0057	Lachnospiraceae
B14 TO LI7	PILOT	DECREASE	Otu0059	Porphyromonadaceae
B14 TO LI7	PILOT	DECREASE	Otu0082	Ruminococcaceae
B14 TO LI7	PILOT	DECREASE	Otu0084	Porphyromonadaceae
B14 TO LI7	PILOT	DECREASE	Otu0094	Rikenellaceae
B14 TO LI7	PILOT	DECREASE	Otu0096	Porphyromonadaceae
B14 TO LI7	PILOT	DECREASE	Otu0097	Porphyromonadaceae
B14 TO LI7	PILOT	DECREASE	Otu0099	Porphyromonadaceae
B14 TO LI7	PILOT	DECREASE	Otu0100	Lachnospiraceae
B14 TO LI7	PILOT	DECREASE	Otu0102	Porphyromonadaceae
B14 TO LI7	PILOT	DECREASE	Otu0116	Porphyromonadaceae
B14 TO LI7	PILOT	DECREASE	Otu0155	Porphyromonadaceae
B14 TO LI7	PILOT	DECREASE	Otu0187	Lachnospiraceae
B14 TO LI7	PILOT	DECREASE	Otu0209	Clostridiales_unclassified
B14 TO LI7	PILOT	DECREASE	Otu0227	Clostridiales_unclassified
B14 TO LI7	VALIDATION 1	INCREASE	Otu0001	Verrucomicrobiaceae
B14 TO LI7	VALIDATION 1	INCREASE	Otu0005	Porphyromonadaceae
B14 TO LI7	VALIDATION 1	INCREASE	Otu0007	Bacteroidaceae
B14 TO LI7	VALIDATION 1	INCREASE	Otu0016	Sutterellaceae
B14 TO LI7	VALIDATION 1	INCREASE	Otu0023	Ruminococcaceae
B14 TO LI7	VALIDATION 1	INCREASE	Otu0035	Lachnospiraceae
B14 TO LI7	VALIDATION 1	INCREASE	Otu0134	Erysipelotrichaceae
B14 TO LI7	VALIDATION 1	DECREASE	Otu0008	Clostridiales_unclassified
B14 TO LI7	VALIDATION 1	DECREASE	Otu0009	Prevotellaceae
B14 TO LI7	VALIDATION 1	DECREASE	Otu0014	Prevotellaceae
B14 TO LI7	VALIDATION 1	DECREASE	Otu0019	Porphyromonadaceae
B14 TO LI7	VALIDATION 1	DECREASE	Otu0021	Rikenellaceae
B14 TO LI7	VALIDATION 1	DECREASE	Otu0032	Bacteroidales_unclassified
B14 TO LI7	VALIDATION 1	DECREASE	Otu0041	Porphyromonadaceae
B14 TO LI7	VALIDATION 1	DECREASE	Otu0045	Lachnospiraceae
B14 TO LI7	VALIDATION 1	DECREASE	Otu0046	Porphyromonadaceae
B14 TO LI7	VALIDATION 1	DECREASE	Otu0049	Porphyromonadaceae

B14 TO LI7	VALIDATION 1	DECREASE	Otu0050	Clostridiales_unclassified
B14 TO LI7	VALIDATION 1	DECREASE	Otu0052	Porphyromonadaceae
B14 TO LI7	VALIDATION 1	DECREASE	Otu0059	Porphyromonadaceae
B14 TO LI7	VALIDATION 1	DECREASE	Otu0082	Ruminococcaceae
B14 TO LI7	VALIDATION 1	DECREASE	Otu0084	Porphyromonadaceae
B14 TO LI7	VALIDATION 1	DECREASE	Otu0094	Rikenellaceae
B14 TO LI7	VALIDATION 1	DECREASE	Otu0096	Porphyromonadaceae
B14 TO LI7	VALIDATION 1	DECREASE	Otu0099	Porphyromonadaceae
B14 TO LI7	VALIDATION 1	DECREASE	Otu0102	Porphyromonadaceae
B14 TO LI7	VALIDATION 1	DECREASE	Otu0111	Lachnospiraceae
B14 TO LI7	VALIDATION 1	DECREASE	Otu0113	Lachnospiraceae
B14 TO LI7	VALIDATION 1	DECREASE	Otu0116	Porphyromonadaceae
B14 TO LI7	VALIDATION 1	DECREASE	Otu0142	Firmicutes_unclassified
B14 TO LI7	VALIDATION 1	DECREASE	Otu0159	Ruminococcaceae
B14 TO LI7	VALIDATION 1	DECREASE	Otu0160	Lachnospiraceae
B14 TO LI7	VALIDATION 1	DECREASE	Otu0161	Ruminococcaceae
B14 TO LI7	VALIDATION 1	DECREASE	Otu0170	Ruminococcaceae
B14 TO LI7	VALIDATION 1	DECREASE	Otu0172	Lachnospiraceae
B14 TO LI7	VALIDATION 1	DECREASE	Otu0193	Ruminococcaceae
B14 TO LI7	VALIDATION 1	DECREASE	Otu0194	Lachnospiraceae
B14 TO LI7	VALIDATION 1	DECREASE	Otu0209	Clostridiales_unclassified
B14 TO LI7	VALIDATION 1	DECREASE	Otu0228	Rikenellaceae
B14 TO LI7	VALIDATION 1	DECREASE	Otu0277	Erysipelotrichaceae
B14 TO LI7	VALIDATION 2	INCREASE	Otu0002	Bacteroidaceae
B14 TO LI7	VALIDATION 2	INCREASE	Otu0016	Sutterellaceae
B14 TO LI7	VALIDATION 2	INCREASE	Otu0022	Porphyromonadaceae
B14 TO LI7	VALIDATION 2	INCREASE	Otu0114	Peptostreptococcaceae
B14 TO LI7	VALIDATION 2	DECREASE	Otu0010	Erysipelotrichaceae
B14 TO LI7	VALIDATION 2	DECREASE	Otu0011	Lachnospiraceae
B14 TO LI7	VALIDATION 2	DECREASE	Otu0012	Lachnospiraceae
B14 TO LI7	VALIDATION 2	DECREASE	Otu0017	Erysipelotrichaceae
B14 TO LI7	VALIDATION 2	DECREASE	Otu0019	Porphyromonadaceae
B14 TO LI7	VALIDATION 2	DECREASE	Otu0021	Rikenellaceae
B14 TO LI7	VALIDATION 2	DECREASE	Otu0036	Lachnospiraceae
B14 TO LI7	VALIDATION 2	DECREASE	Otu0045	Lachnospiraceae
B14 TO LI7	VALIDATION 2	DECREASE	Otu0052	Porphyromonadaceae
B14 TO LI7	VALIDATION 2	DECREASE	Otu0076	Lachnospiraceae
B14 TO LI7	VALIDATION 2	DECREASE	Otu0090	Lachnospiraceae
B14 TO LI7	VALIDATION 2	DECREASE	Otu0100	Lachnospiraceae
B14 TO LI7	VALIDATION 2	DECREASE	Otu0101	Lachnospiraceae
B14 TO LI7	VALIDATION 2	DECREASE	Otu0105	Ruminococcaceae
B14 TO LI7	VALIDATION 2	DECREASE	Otu0128	Lachnospiraceae
B14 TO LI7	VALIDATION 2	DECREASE	Otu0136	Lachnospiraceae
B14 TO LI7	VALIDATION 2	DECREASE	Otu0154	Bacteria_unclassified
B14 TO LI7	VALIDATION 2	DECREASE	Otu0162	Ruminococcaceae
B14 TO LI7	VALIDATION 2	DECREASE	Otu0163	Lachnospiraceae
B14 TO LI7	VALIDATION 2	DECREASE	Otu0169	Firmicutes_unclassified

B14 TO LI7	VALIDATION 2	DECREASE	Otu0187	Lachnospiraceae
B14 TO LI7	VALIDATION 2	DECREASE	Otu0209	Clostridiales_unclassified
B14 TO LI7	VALIDATION 2	DECREASE	Otu0230	Lachnospiraceae
B14 TO LI7	VALIDATION 2	DECREASE	Otu0236	Lachnospiraceae
B14 TO LI7	VALIDATION 2	DECREASE	Otu0279	Lachnospiraceae
B14 TO LI7	REPLETION 1	INCREASE	Otu0001	Verrucomicrobiaceae
B14 TO LI7	REPLETION 1	INCREASE	Otu0005	Porphyromonadaceae
B14 TO LI7	REPLETION 1	INCREASE	Otu0007	Bacteroidaceae
B14 TO LI7	REPLETION 1	INCREASE	Otu0016	Sutterellaceae
B14 TO LI7	REPLETION 1	INCREASE	Otu0022	Porphyromonadaceae
B14 TO LI7	REPLETION 1	INCREASE	Otu0034	Rhodospirillaceae
B14 TO LI7	REPLETION 1	INCREASE	Otu0058	Porphyromonadaceae
B14 TO LI7	REPLETION 1	DECREASE	Otu0009	Prevotellaceae
B14 TO LI7	REPLETION 1	DECREASE	Otu0014	Prevotellaceae
B14 TO LI7	REPLETION 1	DECREASE	Otu0019	Porphyromonadaceae
B14 TO LI7	REPLETION 1	DECREASE	Otu0021	Rikenellaceae
B14 TO LI7	REPLETION 1	DECREASE	Otu0030	Porphyromonadaceae
B14 TO LI7	REPLETION 1	DECREASE	Otu0032	Bacteroidales_unclassified
B14 TO LI7	REPLETION 1	DECREASE	Otu0036	Lachnospiraceae
B14 TO LI7	REPLETION 1	DECREASE	Otu0041	Porphyromonadaceae
B14 TO LI7	REPLETION 1	DECREASE	Otu0045	Lachnospiraceae
B14 TO LI7	REPLETION 1	DECREASE	Otu0046	Porphyromonadaceae
B14 TO LI7	REPLETION 1	DECREASE	Otu0047	Lachnospiraceae
B14 TO LI7	REPLETION 1	DECREASE	Otu0049	Porphyromonadaceae
B14 TO LI7	REPLETION 1	DECREASE	Otu0052	Porphyromonadaceae
B14 TO LI7	REPLETION 1	DECREASE	Otu0059	Porphyromonadaceae
B14 TO LI7	REPLETION 1	DECREASE	Otu0061	Bacteroidales_unclassified
B14 TO LI7	REPLETION 1	DECREASE	Otu0069	Lachnospiraceae
B14 TO LI7	REPLETION 1	DECREASE	Otu0075	Lachnospiraceae
B14 TO LI7	REPLETION 1	DECREASE	Otu0082	Ruminococcaceae
B14 TO LI7	REPLETION 1	DECREASE	Otu0084	Porphyromonadaceae
B14 TO LI7	REPLETION 1	DECREASE	Otu0090	Lachnospiraceae
B14 TO LI7	REPLETION 1	DECREASE	Otu0094	Rikenellaceae
B14 TO LI7	REPLETION 1	DECREASE	Otu0096	Porphyromonadaceae
B14 TO LI7	REPLETION 1	DECREASE	Otu0099	Porphyromonadaceae
B14 TO LI7	REPLETION 1	DECREASE	Otu0100	Lachnospiraceae
B14 TO LI7	REPLETION 1	DECREASE	Otu0102	Porphyromonadaceae
B14 TO LI7	REPLETION 1	DECREASE	Otu0116	Porphyromonadaceae
B14 TO LI7	REPLETION 1	DECREASE	Otu0126	Ruminococcaceae
B14 TO LI7	REPLETION 1	DECREASE	Otu0133	Lachnospiraceae
B14 TO LI7	REPLETION 1	DECREASE	Otu0154	Bacteria_unclassified
B14 TO LI7	REPLETION 1	DECREASE	Otu0155	Porphyromonadaceae
B14 TO LI7	REPLETION 1	DECREASE	Otu0159	Ruminococcaceae
B14 TO LI7	REPLETION 1	DECREASE	Otu0161	Ruminococcaceae
B14 TO LI7	REPLETION 1	DECREASE	Otu0225	Porphyromonadaceae
LI7 TO LI14	PILOT	INCREASE	Otu0002	Bacteroidaceae
LI7 TO LI14	PILOT	INCREASE	Otu0006	Porphyromonadaceae

LI7 TO LI14	PILOT	INCREASE	Otu0018	Lactobacillaceae
LI7 TO LI14	PILOT	INCREASE	Otu0056	Rikenellaceae
LI7 TO LI14	PILOT	INCREASE	Otu0070	Coriobacteriaceae
LI7 TO LI14	PILOT	DECREASE	Otu0001	Verrucomicrobiaceae
LI7 TO LI14	PILOT	DECREASE	Otu0011	Lachnospiraceae
LI7 TO LI14	PILOT	DECREASE	Otu0013	Lachnospiraceae
LI7 TO LI14	PILOT	DECREASE	Otu0015	Lachnospiraceae
LI7 TO LI14	PILOT	DECREASE	Otu0020	Lachnospiraceae
LI7 TO LI14	PILOT	DECREASE	Otu0034	Rhodospirillaceae
LI7 TO LI14	PILOT	DECREASE	Otu0048	Clostridiales_unclassified
LI7 TO LI14	PILOT	DECREASE	Otu0055	Lachnospiraceae
LI7 TO LI14	PILOT	DECREASE	Otu0085	Ruminococcaceae
LI7 TO LI14	PILOT	DECREASE	Otu0122	Lachnospiraceae
LI7 TO LI14	PILOT	DECREASE	Otu0152	Lachnospiraceae
LI7 TO LI14	PILOT	DECREASE	Otu0184	Bacteria_unclassified
LI7 TO LI14	VALIDATION 1	INCREASE	Otu0030	Porphyromonadaceae
LI7 TO LI14	VALIDATION 1	INCREASE	Otu0034	Rhodospirillaceae
LI7 TO LI14	VALIDATION 1	INCREASE	Otu0056	Rikenellaceae
LI7 TO LI14	VALIDATION 1	INCREASE	Otu0058	Porphyromonadaceae
LI7 TO LI14	VALIDATION 1	INCREASE	Otu0091	Clostridiales_unclassified
LI7 TO LI14	VALIDATION 1	INCREASE	Otu0105	Ruminococcaceae
LI7 TO LI14	VALIDATION 1	INCREASE	Otu0108	Lachnospiraceae
LI7 TO LI14	VALIDATION 1	INCREASE	Otu0143	Porphyromonadaceae
LI7 TO LI14	VALIDATION 1	INCREASE	Otu0145	Lachnospiraceae
LI7 TO LI14	VALIDATION 1	DECREASE	Otu0017	Erysipelotrichaceae
LI7 TO LI14	VALIDATION 1	DECREASE	Otu0020	Lachnospiraceae
LI7 TO LI14	VALIDATION 1	DECREASE	Otu0024	Lachnospiraceae
LI7 TO LI14	VALIDATION 1	DECREASE	Otu0025	Porphyromonadaceae
LI7 TO LI14	VALIDATION 1	DECREASE	Otu0043	Ruminococcaceae
LI7 TO LI14	VALIDATION 1	DECREASE	Otu0101	Lachnospiraceae
LI7 TO LI14	VALIDATION 1	DECREASE	Otu0125	Porphyromonadaceae
LI7 TO LI14	VALIDATION 1	DECREASE	Otu0133	Lachnospiraceae
LI7 TO LI14	VALIDATION 1	DECREASE	Otu0138	Lachnospiraceae
LI7 TO LI14	VALIDATION 2	INCREASE	Otu0019	Porphyromonadaceae
LI7 TO LI14	VALIDATION 2	INCREASE	Otu0087	Lachnospiraceae
LI7 TO LI14	VALIDATION 2	DECREASE	Otu0017	Erysipelotrichaceae
LI7 TO LI14	VALIDATION 2	DECREASE	Otu0022	Porphyromonadaceae
LI7 TO LI14	VALIDATION 2	DECREASE	Otu0035	Lachnospiraceae
LI7 TO LI14	VALIDATION 2	DECREASE	Otu0064	Ruminococcaceae
LI7 TO LI14	VALIDATION 2	DECREASE	Otu0077	Erysipelotrichaceae
LI7 TO LI14	VALIDATION 2	DECREASE	Otu0086	Coriobacteriaceae
LI7 TO LI14	VALIDATION 2	DECREASE	Otu0114	Peptostreptococcaceae
LI7 TO LI14	VALIDATION 2	DECREASE	Otu0152	Lachnospiraceae
LI7 TO LI14	REPLETION 1	INCREASE	Otu0025	Porphyromonadaceae
LI7 TO LI14	REPLETION 1	INCREASE	Otu0058	Porphyromonadaceae
LI7 TO LI14	REPLETION 1	INCREASE	Otu0143	Porphyromonadaceae
LI7 TO LI14	REPLETION 1	DECREASE	Otu0003	Erysipelotrichaceae

LI7 TO LI14	REPLETION 1	DECREASE	Otu0007	Bacteroidaceae
LI7 TO LI14	REPLETION 1	DECREASE	Otu0018	Lactobacillaceae
LI7 TO LI14	REPLETION 1	DECREASE	Otu0022	Porphyromonadaceae
LI7 TO LI14	REPLETION 1	DECREASE	Otu0029	Lactobacillaceae
LI7 TO LI14	REPLETION 1	DECREASE	Otu0030	Porphyromonadaceae
LI7 TO LI14	REPLETION 1	DECREASE	Otu0070	Coriobacteriaceae
LI7 TO LI14	REPLETION 2	INCREASE	Otu0001	Verrucomicrobiaceae
LI7 TO LI14	REPLETION 2	INCREASE	Otu0024	Lachnospiraceae
LI7 TO LI14	REPLETION 2	INCREASE	Otu0108	Lachnospiraceae
LI7 TO LI14	REPLETION 2	INCREASE	Otu0109	Lachnospiraceae
LI7 TO LI14	REPLETION 2	INCREASE	Otu0288	Lachnospiraceae
LI7 TO LI14	REPLETION 2	DECREASE	Otu0002	Bacteroidaceae
LI7 TO LI14	REPLETION 2	DECREASE	Otu0014	Prevotellaceae
LI7 TO LI14	REPLETION 2	DECREASE	Otu0016	Sutterellaceae
LI7 TO LI14	REPLETION 2	DECREASE	Otu0019	Porphyromonadaceae
LI7 TO LI14	REPLETION 2	DECREASE	Otu0030	Porphyromonadaceae
LI14 TO R7	VALIDATION 2	INCREASE	Otu0010	Erysipelotrichaceae
LI14 TO R7	VALIDATION 2	INCREASE	Otu0012	Lachnospiraceae
LI14 TO R7	VALIDATION 2	INCREASE	Otu0017	Erysipelotrichaceae
LI14 TO R7	VALIDATION 2	INCREASE	Otu0019	Porphyromonadaceae
LI14 TO R7	VALIDATION 2	INCREASE	Otu0045	Lachnospiraceae
LI14 TO R7	VALIDATION 2	INCREASE	Otu0075	Lachnospiraceae
LI14 TO R7	VALIDATION 2	INCREASE	Otu0087	Lachnospiraceae
LI14 TO R7	VALIDATION 2	INCREASE	Otu0090	Lachnospiraceae
LI14 TO R7	VALIDATION 2	INCREASE	Otu0100	Lachnospiraceae
LI14 TO R7	VALIDATION 2	INCREASE	Otu0105	Ruminococcaceae
LI14 TO R7	VALIDATION 2	INCREASE	Otu0111	Lachnospiraceae
LI14 TO R7	VALIDATION 2	INCREASE	Otu0133	Lachnospiraceae
LI14 TO R7	VALIDATION 2	INCREASE	Otu0145	Lachnospiraceae
LI14 TO R7	VALIDATION 2	INCREASE	Otu0152	Lachnospiraceae
LI14 TO R7	VALIDATION 2	INCREASE	Otu0154	Bacteria_unclassified
LI14 TO R7	VALIDATION 2	INCREASE	Otu0294	Ruminococcaceae
LI14 TO R7	VALIDATION 2	DECREASE	Otu0001	Verrucomicrobiaceae
LI14 TO R7	VALIDATION 2	DECREASE	Otu0024	Lachnospiraceae
LI14 TO R7	VALIDATION 2	DECREASE	Otu0114	Peptostreptococcaceae
LI14 TO R7	REPLETION 1	INCREASE	Otu0007	Bacteroidaceae
LI14 TO R7	REPLETION 1	INCREASE	Otu0012	Lachnospiraceae
LI14 TO R7	REPLETION 1	INCREASE	Otu0019	Porphyromonadaceae
LI14 TO R7	REPLETION 1	INCREASE	Otu0026	Porphyromonadaceae
LI14 TO R7	REPLETION 1	INCREASE	Otu0030	Porphyromonadaceae
LI14 TO R7	REPLETION 1	INCREASE	Otu0036	Lachnospiraceae
LI14 TO R7	REPLETION 1	INCREASE	Otu0042	Desulfovibrionaceae
LI14 TO R7	REPLETION 1	INCREASE	Otu0043	Ruminococcaceae
LI14 TO R7	REPLETION 1	INCREASE	Otu0048	Clostridiales_unclassified
LI14 TO R7	REPLETION 1	INCREASE	Otu0053	Ruminococcaceae
LI14 TO R7	REPLETION 1	INCREASE	Otu0084	Porphyromonadaceae
LI14 TO R7	REPLETION 1	INCREASE	Otu0085	Ruminococcaceae

LI14 TO R7	REPLETION 1	INCREASE	Otu0087	Lachnospiraceae
LI14 TO R7	REPLETION 1	INCREASE	Otu0126	Ruminococcaceae
LI14 TO R7	REPLETION 1	INCREASE	Otu0130	Lachnospiraceae
LI14 TO R7	REPLETION 1	INCREASE	Otu0174	Lachnospiraceae
LI14 TO R7	REPLETION 1	INCREASE	Otu0204	Ruminococcaceae
LI14 TO R7	REPLETION 1	DECREASE	Otu0143	Porphyromonadaceae
LI14 TO R7	REPLETION 2	INCREASE	Otu0010	Erysipelotrichaceae
LI14 TO R7	REPLETION 2	INCREASE	Otu0015	Lachnospiraceae
LI14 TO R7	REPLETION 2	INCREASE	Otu0018	Lactobacillaceae
LI14 TO R7	REPLETION 2	INCREASE	Otu0032	Bacteroidales_unclassified
LI14 TO R7	REPLETION 2	INCREASE	Otu0046	Porphyromonadaceae
LI14 TO R7	REPLETION 2	INCREASE	Otu0050	Clostridiales_unclassified
LI14 TO R7	REPLETION 2	INCREASE	Otu0055	Lachnospiraceae
LI14 TO R7	REPLETION 2	INCREASE	Otu0058	Porphyromonadaceae
LI14 TO R7	REPLETION 2	INCREASE	Otu0072	Porphyromonadaceae
LI14 TO R7	REPLETION 2	INCREASE	Otu0109	Lachnospiraceae
LI14 TO R7	REPLETION 2	INCREASE	Otu0111	Lachnospiraceae
LI14 TO R7	REPLETION 2	INCREASE	Otu0119	Clostridiales_unclassified
LI14 TO R7	REPLETION 2	INCREASE	Otu0153	Ruminococcaceae
LI14 TO R7	REPLETION 2	DECREASE	Otu0005	Porphyromonadaceae
LI14 TO R7	REPLETION 2	DECREASE	Otu0016	Sutterellaceae
LI14 TO R7	REPLETION 2	DECREASE	Otu0023	Ruminococcaceae
LI14 TO R7	REPLETION 2	DECREASE	Otu0028	Bacteroidaceae
LI14 TO R7	REPLETION 2	DECREASE	Otu0034	Rhodospirillaceae
LI14 TO R7	REPLETION 2	DECREASE	Otu0057	Lachnospiraceae
LI14 TO R7	REPLETION 2	DECREASE	Otu0244	Streptococcaceae
R7 TO R14	REPLETION 1	INCREASE	Otu0006	Porphyromonadaceae
R7 TO R14	REPLETION 1	INCREASE	Otu0014	Prevotellaceae
R7 TO R14	REPLETION 1	INCREASE	Otu0015	Lachnospiraceae
R7 TO R14	REPLETION 1	INCREASE	Otu0016	Sutterellaceae
R7 TO R14	REPLETION 1	INCREASE	Otu0018	Lactobacillaceae
R7 TO R14	REPLETION 1	INCREASE	Otu0026	Porphyromonadaceae
R7 TO R14	REPLETION 1	INCREASE	Otu0028	Bacteroidaceae
R7 TO R14	REPLETION 1	INCREASE	Otu0036	Lachnospiraceae
R7 TO R14	REPLETION 1	DECREASE	Otu0004	Bifidobacteriaceae
R7 TO R14	REPLETION 1	DECREASE	Otu0010	Erysipelotrichaceae
R7 TO R14	REPLETION 1	DECREASE	Otu0013	Lachnospiraceae
R7 TO R14	REPLETION 1	DECREASE	Otu0022	Porphyromonadaceae
R7 TO R14	REPLETION 1	DECREASE	Otu0034	Rhodospirillaceae
R7 TO R14	REPLETION 1	DECREASE	Otu0040	Lachnospiraceae
R7 TO R14	REPLETION 1	DECREASE	Otu0085	Ruminococcaceae
R7 TO R14	REPLETION 1	DECREASE	Otu0103	Ruminococcaceae
R7 TO R14	REPLETION 1	DECREASE	Otu0112	Lachnospiraceae
R7 TO R14	REPLETION 1	DECREASE	Otu0198	Bacteroidaceae
R7 TO R14	REPLETION 2	INCREASE	Otu0002	Bacteroidaceae
R7 TO R14	REPLETION 2	INCREASE	Otu0005	Porphyromonadaceae
R7 TO R14	REPLETION 2	INCREASE	Otu0016	Sutterellaceae

R7 TO R14	REPLETION 2	INCREASE	Otu0023	Ruminococcaceae
R7 TO R14	REPLETION 2	INCREASE	Otu0026	Porphyromonadaceae
R7 TO R14	REPLETION 2	INCREASE	Otu0028	Bacteroidaceae
R7 TO R14	REPLETION 2	INCREASE	Otu0030	Porphyromonadaceae
R7 TO R14	REPLETION 2	INCREASE	Otu0032	Bacteroidales_unclassified
R7 TO R14	REPLETION 2	INCREASE	Otu0033	Erysipelotrichaceae
R7 TO R14	REPLETION 2	INCREASE	Otu0034	Rhodospirillaceae
R7 TO R14	REPLETION 2	INCREASE	Otu0042	Desulfovibrionaceae
R7 TO R14	REPLETION 2	INCREASE	Otu0043	Ruminococcaceae
R7 TO R14	REPLETION 2	INCREASE	Otu0054	Ruminococcaceae
R7 TO R14	REPLETION 2	INCREASE	Otu0056	Rikenellaceae
R7 TO R14	REPLETION 2	INCREASE	Otu0060	Ruminococcaceae
R7 TO R14	REPLETION 2	INCREASE	Otu0084	Porphyromonadaceae
R7 TO R14	REPLETION 2	INCREASE	Otu0103	Ruminococcaceae
R7 TO R14	REPLETION 2	INCREASE	Otu0126	Ruminococcaceae
R7 TO R14	REPLETION 2	INCREASE	Otu0161	Ruminococcaceae
R7 TO R14	REPLETION 2	INCREASE	Otu0165	Lachnospiraceae
R7 TO R14	REPLETION 2	INCREASE	Otu0171	Ruminococcaceae
R7 TO R14	REPLETION 2	INCREASE	Otu0181	Ruminococcaceae
R7 TO R14	REPLETION 2	INCREASE	Otu0229	Clostridia_unclassified
R7 TO R14	REPLETION 2	INCREASE	Otu0252	Lachnospiraceae
R7 TO R14	REPLETION 2	INCREASE	Otu0276	Bacteroidaceae
R7 TO R14	REPLETION 2	DECREASE	Otu0001	Verrucomicrobiaceae
R7 TO R14	REPLETION 2	DECREASE	Otu0010	Erysipelotrichaceae
R7 TO R14	REPLETION 2	DECREASE	Otu0011	Lachnospiraceae
R7 TO R14	REPLETION 2	DECREASE	Otu0058	Porphyromonadaceae
R7 TO R14	REPLETION 2	DECREASE	Otu0134	Erysipelotrichaceae
R7 TO R14	REPLETION 2	DECREASE	Otu0153	Ruminococcaceae

GENUS	MEAN FOLD CHANGE	STDEV-1	STDEV-2
Erysipelotrichaceae_unclassified	162	15.6507011	475.68059
Parabacteroides	7.107143	14.2809119	103.65568
Porphyromonadaceae_unclassified	6.273973	52.1946145	79.26222
Bacteroides	6	9.5058462	57.84462
Alloprevotella	5.332103	163.0061349	488.07428
Erysipelotrichaceae_unclassified	9.181818	14.1725478	44.67382
Lachnospiraceae_unclassified	8.923077	19.5092571	166.4678
Porphyromonadaceae_unclassified	6.777778	25.3859104	15.03145
Porphyromonadaceae_unclassified	6.176471	8.5732141	48.5355
Porphyromonadaceae_unclassified	8.333333	8.9271745	22.80412
Parvibacter	UNDEFINED	0.3333333	4.1833
Prevotella	0.004820568	293.4584487	8.0173423
Lachnospiraceae_unclassified	0.096774194	44.2976047	19.784955
Alistipes	0.081784387	100.0838537	15.0037032
Porphyromonadaceae_unclassified	0.029850746	113.4872729	7.3955692
Porphyromonadaceae_unclassified	0.018927445	83.0286095	4.8591266
Porphyromonadaceae_unclassified	0.036363636	22.8369827	1.9220938
Porphyromonadaceae_unclassified	0.058252427	114.4127518	13.6483617
Porphyromonadaceae_unclassified	0.2	57.1358031	11.7839344
Porphyromonadaceae_unclassified	0	101.5797443	0.8819171
Porphyromonadaceae_unclassified	0.111111111	12.660086	2.1794495
Alistipes	0.25	16.8506512	5.4313902
Porphyromonadaceae_unclassified	0	54.0277706	0
Clostridium_sensu_stricto	0	109.7543975	0
Alistipes	0	57.8354947	0.3333333
Porphyromonadaceae_unclassified	0.2125	27.3653268	10.1748601
Lachnospiraceae_unclassified	0	29.4919951	0
Porphyromonadaceae_unclassified	0.096385542	32.8481354	5.7831172
Anaeroplasma	0	56.9583181	0.3333333
Porphyromonadaceae_unclassified	0.006622517	63.0749114	2
Porphyromonadaceae_unclassified	0	17.2803678	0
Ruminococcaceae_unclassified	0	26.3396617	0
Lactobacillus	0.038461538	23.2761203	2.2236107
Ruminococcus	0	63.4803119	0
Lachnospiraceae_unclassified	0	53.5064897	0
Deltaproteobacteria_unclassified	0	29.3404347	4.4190245
Porphyromonadaceae_unclassified	0.058823529	9.797959	0.8660254
Firmicutes_unclassified	0	6.3442888	1.1666667
Porphyromonadaceae_unclassified	0.142857143	4.5552168	1.3944334
Bacteroidales_unclassified	0	13.7153604	0.6666667
Lachnospiraceae_unclassified	0	7.3993243	0.4409586
Porphyromonadaceae_unclassified	0.045454545	4.1264728	1.2360331
Clostridiales_unclassified	0	13.1339255	0
Oscillibacter	0.214285714	14.6695073	1.8559215
Clostridiales_unclassified	0	4.6097722	0
Lachnospiraceae_unclassified	0	8.776547	0

Clostridium_IV	0	5.6960025	0
Clostridia_unclassified	0	5.2783625	0
Firmicutes_unclassified	0	9.9247166	0
Lachnospiraceae_unclassified	0	6.2849025	0
Clostridiales_unclassified	0	1.3642255	0
Clostridiales_unclassified	0	0.6009252	0
Akkermansia	4.105976	443.646619	637.19493
Erysipelotrichaceae_unclassified	58.153846	33.777869	250.52897
Lachnospiraceae_unclassified	3.225	21.007935	33.36598
Lachnospiraceae_unclassified	4.066038	29.566498	111.00651
Erysipelotrichaceae_unclassified	73.473684	117.453726	674.50608
Lachnospiraceae_unclassified	6.416667	7.859884	88.466
Lachnospiraceae_unclassified	31.5	7.11493	260.95202
Lachnospiraceae_unclassified	12.1	4.273952	220.77751
Lachnospiraceae_unclassified	6.785714	4.613988	93.63813
Lachnospiraceae_unclassified	24.444444	13.787676	138.06379
Bacteroides	0.52941176	530.35485	310.3747556
Alistipes	0.02512315	240.003796	169.1160351
Lactobacillus	0.04651163	204.585353	21.8492309
Porphyromonadaceae_unclassified	0.07696391	282.18475	105.23223
Turicibacter	0.00286944	208.414651	79.6986686
Lachnospiraceae_unclassified	0.08791209	31.130728	6.9673843
Lachnospiraceae_unclassified	0.2826087	21.931459	6.3456022
Lachnospiraceae_unclassified	0	64.017012	15.9024806
Eisenbergiella	0	89.938559	7.6040925
Lachnospiraceae_unclassified	0	41.878793	6.9153613
Lachnospiraceae_unclassified	0	39.766541	5.3427001
Bacteria_unclassified	0	16.425252	2.2730303
Lachnospiraceae_unclassified	0	7.781031	0.3162278
Bacteria_unclassified	0	4.24395	0
Clostridiales_unclassified	3	154.784079	401.3231588
Acetatifactor	3.083333	16.9246697	42.4444081
Porphyromonadaceae_unclassified	1.623529	44.2910199	58.7262955
Lachnospiraceae_unclassified	UNDEFINED	11.1592015	18.8731143
Lachnospiraceae_unclassified	UNDEFINED	2.9486343	10.0719633
Lachnospiraceae_unclassified	2.533333	15.4497393	25.3678493
Firmicutes_unclassified	UNDEFINED	1.1666667	7.1414284
Lachnospiraceae_unclassified	2.833333	2.6977357	17.9961416
Firmicutes_unclassified	UNDEFINED	1.3017083	1.8708287
Bacteria_unclassified	UNDEFINED	0.7071068	0.6009252
Porphyromonadaceae_unclassified	0	7.395569	1.0137938
Lactobacillus	0	39.003561	0.8819171
Porphyromonadaceae_unclassified	0.5	1.922094	0.5270463
Erysipelotrichaceae_unclassified	0	69.21705	10.3802697
Porphyromonadaceae_unclassified	0.4571429	48.535497	27.0714076
Lachnospiraceae_unclassified	0.2380952	16.699135	8.3066239
Olsenella	0.3333333	4.609772	3.0413813

Porphyromonadaceae_unclassified	0.6470588	10.17486	4.6904158
Lachnospiraceae_unclassified	0.125	11.815715	6.827233
Lachnospiraceae_unclassified	0.1818182	42.326443	1.6914819
Akkermansia	1.223365	637.1949292	605.307521
Erysipelotrichaceae_unclassified	2.18254	250.5289737	214.935455
Lachnospiraceae_unclassified	4.528926	220.777515	200.53678
Lachnospiraceae_unclassified	4.28777	99.9504877	88.858189
Lachnospiraceae_unclassified	1.75	1.9119507	4.31406
Ruminococcaceae_unclassified	UNDEFINED	0.6324555	1.414214
Bacteria_unclassified	4.666667	3.6224608	3.689324
Bifidobacterium	0	13.68048	1.013794
Erysipelotrichaceae_unclassified	0.58810888	674.50608	383.694866
Porphyromonadaceae_unclassified	0.2642369	235.48475	54.922673
Lactobacillus	0.09090909	21.84923	1.922094
Clostridium_XVIII	0.125	54.09672	12.248583
Clostridium_sensu_stricto	0.15384615	41.30967	2.297341
Ruminococcaceae_unclassified	0.30769231	27.74907	2.697736
Lachnospiraceae_unclassified	0.31325301	31.67912	24.046829
Lachnospiraceae_unclassified	0.36792453	75.49069	85.347818
Lachnospiraceae_unclassified	0	15.07942	2.862594
Lachnospiraceae_unclassified	3.125749	176.4573918	267.061458
Lachnospiraceae_unclassified	5.015075	305.4109981	265.50725
Lachnospiraceae_unclassified	2.373494	179.7133829	321.072162
Lachnospiraceae_unclassified	3.75	23.3666429	37.873474
Clostridium_XlVa	28.666667	18.9094333	47.12395
Lachnospiraceae_unclassified	7.545455	46.2245726	203.875343
Lachnospiraceae_unclassified	6.95	23.2369438	82.200568
Ruminococcaceae_unclassified	2.5	25.0812015	30.463822
Lachnospiraceae_unclassified	2.769231	16.0886433	39.47939
Lachnospiraceae_unclassified	3.8	8.8969408	39.995139
Acetatifactor	2.15	14.3313952	10.327956
Lachnospiraceae_unclassified	2.214286	4.532598	22.18508
Lachnospiraceae_unclassified	4.2	2.4129281	5.641119
Clostridium_IV	6.666667	1.6996732	4.988877
Lachnospiraceae_unclassified	UNDEFINED	0	4.110961
Clostridium_IV	UNDEFINED	0.8498366	1.969207
Lachnospiraceae_unclassified	UNDEFINED	0	1.159502
Akkermansia	0.60281871	866.419894	801.6546014
Erysipelotrichaceae_unclassified	0.03196347	989.42668	11.4523166
Prevotella	0.2173913	165.760336	60.8003107
Porphyromonadaceae_unclassified	0.07272727	7.015063	2.0655911
Ruminococcaceae_unclassified	0.32801822	77.361776	34.3001458
Porphyromonadaceae_unclassified	0.38461538	9.62693	10.2219807
Ruminococcaceae_unclassified	0.3	3.583915	1.2292726
Ruminococcaceae_unclassified	0.33333333	2.960856	0.6324555
Akkermansia	1.42369	582.998609	573.90439
Bifidobacterium	4.15735	117.262715	324.56719

Parabacteroides	7.067308	62.81861	347.20207
Acetatifactor	9.448276	9.534149	66.29501
Porphyromonadaceae_unclassified	24	2.330951	63.65925
Alloprevotella	0	309.586463	0
Prevotella	0	158.408193	3.2787193
Lachnospiraceae_unclassified	0.04975124	171.172947	8.4113019
Porphyromonadaceae_unclassified	0	35.32091	0
Alistipes	0	18.015117	0.3333333
Bacteroidales_unclassified	0	65.825105	0
Erysipelotrichaceae_unclassified	0	5.130519	0
Porphyromonadaceae_unclassified	0	74.803149	0
Porphyromonadaceae_unclassified	0	39.325564	0
Clostridiales_unclassified	0.47368421	14.719601	5.2148293
Porphyromonadaceae_unclassified	0	72.456577	0
Porphyromonadaceae_unclassified	0	2.624669	0
Clostridium_XlVb	0.01315789	49.29503	7.2762933
Barnesiella	0	40.458621	0
Ruminococcaceae_unclassified	0	8.591986	0
Porphyromonadaceae_unclassified	0	6.310485	0
Alistipes	0	20.024707	0
Porphyromonadaceae_unclassified	0	4.570436	0
Porphyromonadaceae_unclassified	0	13.502675	0
Porphyromonadaceae_unclassified	0	8.680118	0
Lachnospiraceae_unclassified	0	7.930252	0.4409586
Porphyromonadaceae_unclassified	0	9.045564	0
Porphyromonadaceae_unclassified	0	6.289321	0
Porphyromonadaceae_unclassified	0	7.763161	0
Lachnospiraceae_unclassified	0	14.114217	0
Clostridiales_unclassified	0	2.983287	0
Clostridiales_unclassified	0	3.573047	0
Akkermansia	1.598551	422.5798741	231.099546
Parabacteroides	4.723077	66.8270986	176.298752
Bacteroides	4.008065	85.1043477	208.881811
Parasutterella	4.967742	40.224716	72.936807
Ruminococcaceae_unclassified	1.685393	24.0768446	43.988635
Lachnospiraceae_unclassified	UNDEFINED	1.3944334	6.948221
Erysipelotrichaceae_unclassified	UNDEFINED	0.3333333	7.333333
Clostridiales_unclassified	0.02790015	401.3231588	64.1445849
Alloprevotella	0	686.314068	0
Prevotella	0	12.0519708	0.3333333
Porphyromonadaceae_unclassified	0	19.0204568	0
Alistipes	0	13.5503177	0.3333333
Bacteroidales_unclassified	0	34.6305902	0
Porphyromonadaceae_unclassified	0	39.5087895	0
Lachnospiraceae_unclassified	0.02222222	97.0405127	0.8660254
Porphyromonadaceae_unclassified	0	12.3400882	0
Porphyromonadaceae_unclassified	0	27.0714076	0

Clostridiales_unclassified	0	39.6326885	0
Porphyromonadaceae_unclassified	0	0.8660254	0
Barnesiella	0	7.5406307	0
Ruminococcaceae_unclassified	0	13.611678	0
Porphyromonadaceae_unclassified	0	4.6904158	0.3333333
Alistipes	0	57.9676634	0
Porphyromonadaceae_unclassified	0	6.2070748	0
Porphyromonadaceae_unclassified	0	28.5482048	0
Porphyromonadaceae_unclassified	0	9.9749687	0
Lachnospiraceae_unclassified	0.1	6.2472216	1.5811388
Lachnospiraceae_unclassified	0	6.827233	0
Porphyromonadaceae_unclassified	0	7.6648549	0
Firmicutes_unclassified	0	7.1414284	0.6666667
Ruminococcaceae_unclassified	0	12.1151879	0.7071068
Lachnospiraceae_unclassified	0	24.109703	1.118034
Oscillibacter	0	19.6327391	2.3511227
Ruminococcaceae_unclassified	0	2.1213203	0
Lachnospiraceae_unclassified	0	3.9510899	0.7264832
Ruminococcaceae_unclassified	0	6.9061165	0
Lachnospiraceae_unclassified	0	3.2446537	0
Clostridiales_unclassified	0	2.8333333	0
Alistipes	0	3.8078866	0.3333333
Erysipelotrichaceae_unclassified	0	1.5365907	0
Bacteroides	2.153846	299.75115	709.56191
Parasutterella	5.303797	33.33583	123.84062
Porphyromonadaceae_unclassified	6.137931	54.92267	150.87311
Romboutsia	7.3	16.52271	46.60949
Erysipelotrichaceae_unclassified	0.442424242	214.9354554	191.889291
Lachnospiraceae_unclassified	0.32183908	28.785606	32.2722791
Lachnospiraceae_unclassified	0.045822102	264.3697432	71.454531
Erysipelotrichaceae_unclassified	0.040194884	383.6948657	30.7652401
Porphyromonadaceae_unclassified	0.002155172	116.5233644	14.0336896
Alistipes	0	25.1186075	4.5552168
Clostridium_XlVa	0.057142857	29.3683503	3.4920545
Lachnospiraceae_unclassified	0.003649635	200.5367797	8.0156098
Porphyromonadaceae_unclassified	0	59.0063556	0
Lachnospiraceae_unclassified	0	88.8581891	0
Lachnospiraceae_unclassified	0	18.9831797	2.2973415
Lachnospiraceae_unclassified	0	24.0468293	0.7071068
Lachnospiraceae_unclassified	0	24.3897747	0
Ruminococcaceae_unclassified	0	3.2058973	0.3333333
Lachnospiraceae_unclassified	0	85.3478178	0
Lachnospiraceae_unclassified	0	39.3541612	0
Bacteria_unclassified	0	6.5595562	0
Oscillibacter	0	3.1269438	0
Lachnospiraceae_unclassified	0	30.359421	0
Firmicutes_unclassified	0	8.3732378	0

Lachnospiraceae_unclassified	0	10.252371	0
Clostridiales_unclassified	0	0.7071068	0
Lachnospiraceae_unclassified	0	4.9441323	0
Lachnospiraceae_unclassified	0	2.0883273	0
Blautia	0	0.8819171	0
Akkermansia	2.278427	801.654601	976.78214
Parabacteroides	26.4	34.544014	345.25039
Bacteroides	6.86087	38.271109	243.47049
Parasutterella	58.75	3.425395	71.62875
Porphyromonadaceae_unclassified	20.25	2.065591	71.99846
Rhodospirillaceae_unclassified	14.5	7.498889	55.85338
Porphyromonadaceae_unclassified	11.666667	1.414214	34.60186
Alloprevotella	0	376.085156	0
Prevotella	0.04736842	60.800311	5.2535702
Porphyromonadaceae_unclassified	0	16.63497	4.3410188
Alistipes	0	12.980755	0
Porphyromonadaceae_unclassified	0.12269939	43.647706	9.6729864
Bacteroidales_unclassified	0	81.400519	0
Clostridium_XlVa	0.03488372	47.12395	1.3540064
Porphyromonadaceae_unclassified	0	28.797376	0
Lachnospiraceae_unclassified	0	203.875343	0.7071068
Porphyromonadaceae_unclassified	0	31.162656	0
Lachnospiraceae_unclassified	0	82.200568	0.6749486
Porphyromonadaceae_unclassified	0	50.376361	0
Porphyromonadaceae_unclassified	0	21.114503	0
Barnesiella	0	36.900617	0
Bacteroidales_unclassified	0	50.210225	0
Lachnospiraceae_unclassified	0	39.47939	5.626327
Lachnospiraceae_unclassified	0	39.995139	0.3162278
Ruminococcaceae_unclassified	0	63.37446	0
Porphyromonadaceae_unclassified	0	7.192589	0.4830459
Lachnospiraceae_unclassified	0	41.247896	0
Alistipes	0	18.709772	0
Porphyromonadaceae_unclassified	0	10.454133	0
Porphyromonadaceae_unclassified	0	8.329999	0
Lachnospiraceae_unclassified	0	14.621141	0.3162278
Porphyromonadaceae_unclassified	0	9.342852	0
Porphyromonadaceae_unclassified	0	10.221981	0
Ruminococcaceae_unclassified	0	6.961002	0.6749486
Lachnospiraceae_unclassified	0	2.068279	2.8460499
Bacteria_unclassified	0	4.195235	0
Porphyromonadaceae_unclassified	0	4.863698	0
Ruminococcaceae_unclassified	0	21.072362	0
Oscillibacter	0	15.970807	0
Porphyromonadaceae_unclassified	0	3.979112	0
Bacteroides	1.536726	426.556203	301.94231
Porphyromonadaceae_unclassified	1.636187	138.274887	208.09306

Lactobacillus	3.22	34.325646	70.4462
Alistipes	3.928571	9.588767	24.66757
Olsenella	2.458333	5.206833	11.77049
Akkermansia	0.76348436	573.904391	375.7028377
Lachnospiraceae_unclassified	0.29444444	71.869867	25.1548537
Lachnospiraceae_unclassified	0.08333333	38.969361	3.4576807
Lachnospiraceae_unclassified	0.15	8.411302	2.9888682
Acetatifactor	0.2080292	66.295006	36.2780926
Rhodospirillaceae_unclassified	0.31578947	23.504137	3.2659863
Clostridiales_unclassified	0.27777778	5.214829	14.1895893
Lachnospiraceae_unclassified	0	3.278719	0.6749486
Ruminococcaceae_unclassified	0.26923077	13.122288	3.8355066
Lachnospiraceae_unclassified	0.125	5.517648	3.3993463
Lachnospiraceae_unclassified	0	2.061553	0.3162278
Bacteria_unclassified	0.5	2.934469	0.5163978
Porphyromonadaceae_unclassified	5.75	1.8027756	5.548488
Rhodospirillaceae_unclassified	2.847059	93.8669271	94.875482
Alistipes	1.642857	29.9016908	33.978985
Porphyromonadaceae_unclassified	3.5	11.3773654	12.78322
Clostridiales_unclassified	0.5	2.7838822	22.664872
Ruminococcaceae_unclassified	1.625	6.9542153	13.815106
Lachnospiraceae_unclassified	UNDEFINED	1.6583124	13.88473
Porphyromonadaceae_unclassified	2	2.6457513	4.242641
Lachnospiraceae_unclassified	UNDEFINED	0.7071068	12.31651
Erysipelotrichaceae_unclassified	0	4.092676	0.7440238
Acetatifactor	0.5168067	163.296339	94.8468691
Lachnospiraceae_unclassified	0.62	55.473217	15.7003185
Porphyromonadaceae_unclassified	0.5	88.69611	48.4797454
Oscillibacter	0.5637255	42.117231	52.5927752
Lachnospiraceae_unclassified	0	9.974969	0
Porphyromonadaceae_unclassified	0	11.945478	10.377689
Lachnospiraceae_unclassified	0	4.927248	0
Lachnospiraceae_unclassified	0.25	34.590863	5.2915026
Porphyromonadaceae_unclassified	32	14.0336896	74.180149
Lachnospiraceae_unclassified	UNDEFINED	0.6666667	1.013794
Erysipelotrichaceae_unclassified	0	30.76524	0.8660254
Porphyromonadaceae_unclassified	0.3651685	150.873107	86.0494044
Lachnospiraceae_unclassified	0.1175523	293.59231	375.3698176
Ruminococcaceae_unclassified	0.1818182	9.475114	4.9356976
Turicibacter	0.25	2.5	1.2360331
Enterorhabdus	0.2222222	4.558265	3.5862391
Romboutsia	0.4657534	46.609489	16.8159977
Lachnospiraceae_unclassified	0	1.732051	0
Porphyromonadaceae_unclassified	25.333333	28.347251	36.237642
Porphyromonadaceae_unclassified	2.485714	34.601863	69.128624
Porphyromonadaceae_unclassified	2.272727	3.973523	7.334091
Erysipelotrichaceae_unclassified	0.12284483	460.608643	156.462846

Bacteroides	0.26742712	243.470486	284.905462
Lactobacillus	0.35714286	19.922349	5.849976
Porphyromonadaceae_unclassified	0.02469136	71.998457	12.38996
Lactobacillus	0	58.952335	2.394438
Porphyromonadaceae_unclassified	0.15	9.672986	1.414214
Olsenella	0.22891566	27.002881	7.607745
Akkermansia	1.498119	619.590949	1186.144473
Lachnospiraceae_unclassified	3.241379	16.718586	170.779943
Lachnospiraceae_unclassified	UNDEFINED	11.8865	33.112603
Lachnospiraceae_unclassified	UNDEFINED	2.211083	21.782766
Lachnospiraceae_unclassified	UNDEFINED	0	2.643651
Bacteroides	0.4546875	259.311567	179.3980367
Prevotella	0	6.397916	0.3162278
Parasutterella	0.2872928	126.689647	62.6045792
Porphyromonadaceae_unclassified	0	51.083918	0
Porphyromonadaceae_unclassified	0.1333333	12.410121	5.6999025
Erysipelotrichaceae_unclassified	5.021352	134.2017552	492.7517631
Lachnospiraceae_unclassified	157.5	56.6038868	93.5350202
Erysipelotrichaceae_unclassified	UNDEFINED	0.8660254	114.1520915
Porphyromonadaceae_unclassified	15.5	74.1801486	138.6856157
Lachnospiraceae_unclassified	UNDEFINED	0.5	11.5974135
Lachnospiraceae_unclassified	4.833333	8.2310388	10.173495
Lachnospiraceae_unclassified	4	1.0137938	2.5884358
Lachnospiraceae_unclassified	UNDEFINED	0.6666667	25.4715528
Lachnospiraceae_unclassified	UNDEFINED	0	3.7013511
Ruminococcaceae_unclassified	UNDEFINED	0	2.1908902
Lachnospiraceae_unclassified	UNDEFINED	1.4529663	8.4557673
Lachnospiraceae_unclassified	UNDEFINED	0.3333333	2.5884358
Lachnospiraceae_unclassified	UNDEFINED	0	24.7123451
Lachnospiraceae_unclassified	UNDEFINED	0	0.8944272
Bacteria_unclassified	UNDEFINED	0	1.1401754
Ruminococcaceae_unclassified	UNDEFINED	0.3333333	5.1185936
Akkermansia	0.69586777	537.49	432.926899
Lachnospiraceae_unclassified	0.25903614	236.6631	45.877009
Romboutsia	0.05882353	16.816	1.581139
Bacteroides	5.35545	284.9054619	357.622287
Lachnospiraceae_unclassified	UNDEFINED	18.3412104	231.057785
Porphyromonadaceae_unclassified	UNDEFINED	1.5811388	41.245606
Porphyromonadaceae_unclassified	UNDEFINED	8.694826	62.833112
Porphyromonadaceae_unclassified	6.666667	1.4142136	5.01996
Clostridium_XlVa	UNDEFINED	2.6997942	12.316655
Desulfovibrionaceae_unclassified	10.068966	23.8348484	72.840923
Oscillibacter	22.8	13.4313067	52.614637
Clostridiales_unclassified	4.058824	15.195394	21.60324
Ruminococcaceae_unclassified	47.5	5.5226805	99.843878
Porphyromonadaceae_unclassified	UNDEFINED	0	3.535534
Ruminococcaceae_unclassified	7.6	12.6951434	5.215362

Lachnospiraceae_unclassified	UNDEFINED	1.0801234	7.661593
Ruminococcaceae_unclassified	UNDEFINED	0.421637	6.745369
Lachnospiraceae_unclassified	UNDEFINED	0	20.825465
Lachnospiraceae_unclassified	UNDEFINED	0	2.302173
Ruminococcaceae_unclassified	UNDEFINED	0.3162278	1.48324
Porphyromonadaceae_unclassified	0.32	7.334091	1.140175
Erysipelotrichaceae_unclassified	5.277778	124.6813271	353.183097
Lachnospiraceae_unclassified	7.068966	100.0422688	336.007738
Lactobacillus	5.880184	224.8777446	154.920948
Bacteroidales_unclassified	UNDEFINED	0	56.756497
Porphyromonadaceae_unclassified	UNDEFINED	0	46.202814
Clostridiales_unclassified	UNDEFINED	0	147.13939
Lachnospiraceae_unclassified	6	4.9227364	22.799123
Porphyromonadaceae_unclassified	13.47619	17.7203587	141.596963
Porphyromonadaceae_unclassified	UNDEFINED	0.6749486	4.878524
Lachnospiraceae_unclassified	8.666667	21.7827659	83.700657
Lachnospiraceae_unclassified	25	1.8737959	29.359837
Clostridiales_unclassified	25	5.0376361	16.516658
Butyricicoccus	6.666667	1.3374935	13.982131
Parabacteroides	0.0412637	417.230072	91.8433449
Parasutterella	0.05128205	62.604579	3.5355339
Ruminococcaceae_unclassified	0.18367347	68.293566	5.4037024
Bacteroides	0	18.416176	3.4928498
Rhodospirillaceae_unclassified	0.0952381	61.744995	4.4384682
Clostridium_XlVb	0.25	27.958104	0.5477226
Streptococcus	0	1.791957	0
Porphyromonadaceae_unclassified	1.966667	152.601769	115.44133
Prevotella	8.714286	8.378544	22.98478
Lachnospiraceae_unclassified	UNDEFINED	95.48403	115.37027
Parasutterella	7.3	18.201648	59.43652
Lactobacillus	2.333333	3.847077	11.33578
Porphyromonadaceae_unclassified	1.720238	62.833112	60.23039
Bacteroides	7.714286	11.045361	44.50056
Clostridium_XlVa	1.727273	12.316655	13.27403
Bifidobacterium	0.15254237	243.71192	16.3920713
Erysipelotrichaceae_unclassified	0	8.018728	0
Lachnospiraceae_unclassified	0	401.325554	113.5922533
Porphyromonadaceae_unclassified	0	14.380542	0.4472136
Rhodospirillaceae_unclassified	0.07317073	70.467723	5.1768716
Lachnospiraceae_unclassified	0.04022989	253.125463	58.7494681
Ruminococcaceae_unclassified	0.42105263	5.215362	7.0922493
Ruminococcaceae_unclassified	0.5	5.43139	10.1094016
Dorea	0	4.582576	1.3416408
Bacteroides	0	2.880972	0
Bacteroides	11.848837	120.9681776	271.029365
Parabacteroides	16	91.8433449	202.847028
Parasutterella	29	3.5355339	18.743888

Ruminococcaceae_unclassified	6.666667	5.4037024	32.01432
Porphyromonadaceae_unclassified	73.125	3.2093613	66.760143
Bacteroides	UNDEFINED	3.4928498	14.0119
Porphyromonadaceae_unclassified	4.375	2.5884358	5.477226
Bacteroidales_unclassified	2.074713	56.7564974	29.320073
Erysipelotrichaceae_unclassified	UNDEFINED	0.8944272	53.385391
Rhodospirillaceae_unclassified	6.1	4.4384682	27.207536
Desulfovibrionaceae_unclassified	14.3125	8.093207	29.170476
Oscillibacter	13.166667	3.1144823	38.509739
Oscillibacter	27.5	3.8340579	17.951323
Alistipes	7.3	26.387497	101.700541
Ruminococcaceae_unclassified	12.25	3.9623226	24.097026
Porphyromonadaceae_unclassified	UNDEFINED	0.4472136	8.062258
Ruminococcaceae_unclassified	8	1.5165751	3.304038
Ruminococcaceae_unclassified	UNDEFINED	0	5.909033
Oscillibacter	UNDEFINED	0.4472136	8.812869
Lachnospiraceae_unclassified	UNDEFINED	0.8944272	51.376875
Oscillibacter	UNDEFINED	1.3416408	17.056279
Pseudoflavonifractor	UNDEFINED	3.9370039	12.463279
Clostridia_unclassified	UNDEFINED	0	6.683313
Lachnospiraceae_unclassified	UNDEFINED	0	1
Bacteroides	UNDEFINED	0	1.707825
Akkermansia	0.55799743	924.911996	635.132729
Erysipelotrichaceae_unclassified	0.375	353.183097	33.180064
Lachnospiraceae_unclassified	0.24395161	64.766504	14.952703
Porphyromonadaceae_unclassified	0.05477032	141.596963	16.660832
Erysipelotrichaceae_unclassified	0	5.958188	0
Butyricicoccus	0.3	13.982131	2.217356
